# WEE1 inhibition enhances the antitumor immune response to PD-L1 blockade by the concomitant activation of STING and STAT1 pathways in SCLC

**DOI:** 10.1016/j.celrep.2022.110814

**Published:** 2022-05-17

**Authors:** Hirokazu Taniguchi, Rebecca Caeser, Shweta S. Chavan, Yingqian A. Zhan, Andrew Chow, Parvathy Manoj, Fathema Uddin, Hidenori Kitai, Rui Qu, Omar Hayatt, Nisargbhai S. Shah, Álvaro Quintanal Villalonga, Viola Allaj, Evelyn M. Nguyen, Joseph Chan, Adam O. Michel, Hiroshi Mukae, Elisa de Stanchina, Charles M. Rudin, Triparna Sen

**Affiliations:** 1 Department of Medicine, Thoracic Oncology Service, Memorial Sloan Kettering Cancer Center, Mortimer B. Zuckerman Research Center, Office: Z1701, 417 E 68th St, New York, NY 10065, USA; 2 Marie-Josée and Henry R. Kravis Center for Molecular Oncology, Memorial Sloan Kettering Cancer Center, New York, NY 10065, USA; 3 Center for Epigenetics Research, Memorial Sloan Kettering Cancer Center, New York, NY, 10065, USA; 4 Program in Molecular Pharmacology, Memorial Sloan Kettering Cancer Center, New York, NY 10065, USA; 5 Antitumor Assessment Core, Memorial Sloan Kettering Cancer Center, New York, NY 10065, USA; 6 Cancer Biology Program, Louis V. Gerstner Jr. Graduate School of Biomedical Sciences, Memorial Sloan Kettering Cancer Center, New York, NY 10065, USA; 7 Program for Computational and Systems Biology, Memorial Sloan Kettering Cancer Center, New York, NY 10065, USA; 8 Parker Institute for Cancer Immunotherapy, Memorial Sloan Kettering Cancer Center, New York, NY 10065, USA; 9 Drug Safety and Pharmacometrics, Regeneron Pharmaceuticals, Tarrytown, NY 10591, USA; 10 Laboratory of Comparative Pathology, Memorial Sloan Kettering Cancer Center, New York, NY 10065, USA; 11 Department of Respiratory Medicine, Nagasaki University Graduate School of Biomedical Sciences, Nagasaki, 852-8501, Japan; 12 Weill Cornell Medical College, New York, NY 10065, USA; 13 Lead contact

## Abstract

Small cell lung cancers (SCLCs) have high mutational burden but are relatively unresponsive to immune checkpoint blockade (ICB). Using SCLC models, we demonstrate that inhibition of WEE1, a G2/M checkpoint regulator induced by DNA damage, activates the STING-TBK1-IRF3 pathway, which increases type I interferons (IFN-α and IFN-β) and pro-inflammatory chemokines (CXCL10 and CCL5), facilitating an immune response via CD8^+^ cytotoxic T cell infiltration. We further show that WEE1 inhibition concomitantly activates the STAT1 pathway, increasing IFN-γ and PD-L1 expression. Consistent with these findings, combined WEE1 inhibition (AZD1775) and PD-L1 blockade causes remarkable tumor regression, activation of type I and II interferon pathways, and infiltration of cytotoxic T cells in multiple immunocompetent SCLC genetically engineered mouse models, including an aggressive model with stabilized *MYC*. Our study demonstrates cell-autonomous and immune-stimulating activity of WEE1 inhibition in SCLC models. Combined inhibition of WEE1 plus PD-L1 blockade represents a promising immunotherapeutic approach in SCLC.

## In brief

Taniguchi et al. show that WEE1 inhibition activates cGAS-STING, which increases type I interferons and pro-inflammatory chemokines, facilitating an immune response via CD8+ cytotoxic T cell infiltration in SCLC. WEE1 inhibition concomitantly activates STAT1 signaling, increasing IFN-γ and PD-L1 expression, and enhances the efficacy of PD-L1 blockade in SCLC mouse models.

## Graphical Abstract

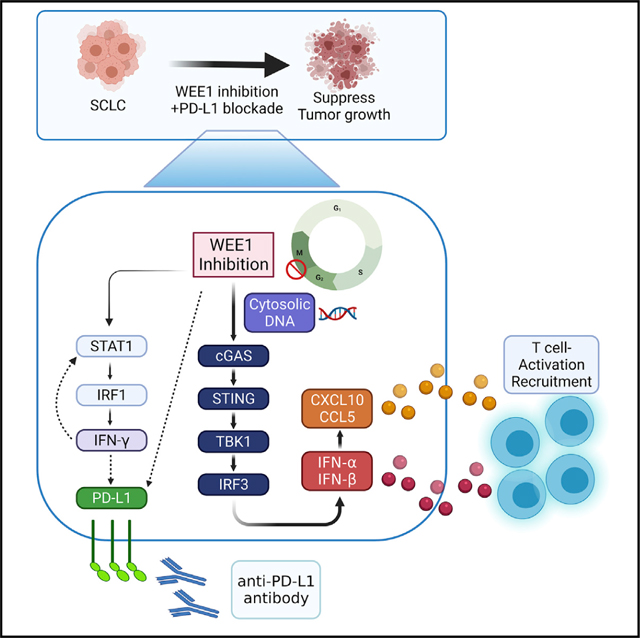

## INTRODUCTION

Small cell lung cancer (SCLC) is a poorly immunogenic, high-grade neuroendocrine carcinoma arising in the lung. Immune checkpoint blockade (ICB) added to chemotherapy improves survival and is now the standard upfront therapy (Horn et al., 2018; Liu et al., 2021) for SCLC but leads to a modest increase in overall survival (OS) and progression-free survival (PFS) (Paz-Ares et al., 2019). Despite a relatively high tumor mutation burden (TMB), ICB is ineffective in most patients with SCLC, regardless of whether the regimen targets the PD-1/PD-L1 axis alone or is combined with anti-CTLA-4 (Antonia et al., 2016; Ott et al., 2017). These modest benefits underscore the critical need to identify pathways and targets that can durably enhance the antitumor responses of ICB in SCLC.

The DNA damage response (DDR) pathway is frequently altered in cancer and is heavily regulated to protect cells against genotoxic damage and intrinsic or extrinsic DNA damage. The DDR pathway regulates entry into mitosis after completion of DNA replication and delays the onset of mitosis if DNA damage is detected. Oncogenes and tumor suppressors associated with replication stress are commonly altered in SCLC (*MYC*, *TP53*, *RB1)* (George et al., 2015; Rudin et al., 2012, 2019; Sen et al., 2018; Taniguchi et al., 2020). As a result, SCLC cells have a vulnerability in DDR pathways and cell-cycle checkpoints (Foy et al., 2017). We and others have shown that DDR components (CHK1, ATR, WEE1, PARP, ATM) are overexpressed in SCLC and that replication-stress-response inhibitors are active in preclinical models of SCLC (Byers and Rudin, 2015; Byers et al., 2012; Dammert et al., 2019; Sen et al., 2017a, 2017b, 2018; Taniguchi et al., 2020).

WEE1 is a tyrosine kinase that regulates cell-cycle progression mainly by phosphorylating and inhibiting cyclin-dependent kinase (CDK) 1 (Do et al., 2013). Inhibition of WEE1 sensitizes ovarian, colon, cervical, osteosarcoma, glioblastoma, and lung cancer cells to DNA damage by irradiation and topoisomerase inhibition (PosthumaDeBoer et al., 2011; Wang et al., 2001, 2004). A selective and potent small-molecule WEE1 inhibitor, AZD1775, has been tested in both preclinical and clinical studies across disease types. AZD1775 was tolerable both as monotherapy and in combination with chemotherapy and had better clinical efficacy in patients with ovarian cancer whose tumors demonstrated mutant versus wildtype *TP53* (Leijen et al., 2016a, 2016b; Oza et al., 2020). A recent study also demonstrated that treatment with AZD1775 was associated with PFS improvement in patients with metastatic colorectal cancer that had *TP53* and *RAS* mutations (Seligmann et al., 2021). Loss of *TP53* disrupts the G1-S cell-cycle checkpoint; as a result, most SCLCs are dependent on G2-M cell-cycle checkpoint regulators, including WEE1. Preclinically, single-agent AZD1775 treatment induced cell-cycle arrest and apoptosis in various SCLC cell lines (Sen et al., 2017a). Mechanistically, other cell-cycle or DDR inhibitors (targeting PARP, CHK1, or CDK7) in SCLC elicited antitumor responses through immune reengagement and direct antitumor effects (Sen et al., 2019a, 2019b; Zhang et al., 2020). Although WEE1 inhibition showed a direct antitumor effect in SCLC (Sen et al., 2017a), the role of the immune response in the antitumor response to WEE1 inhibition in SCLC is currently unknown.

In the current study, we found that WEE1 inhibition increased tumor-infiltrating T cells by activating the innate antitumor immune-response pathway in SCLC models. We further demonstrated that anti-PD-L1 antibody enhanced the antitumor effect of pharmacological WEE1 inhibition. Concurrent treatment with a WEE1 inhibitor and ICB is a potentially effective treatment for SCLC and is worthy of further investigation.

## RESULTS

### WEE1 inhibition induced cell-cycle arrest and cell apoptosis in SCLC

**Figure 1.**
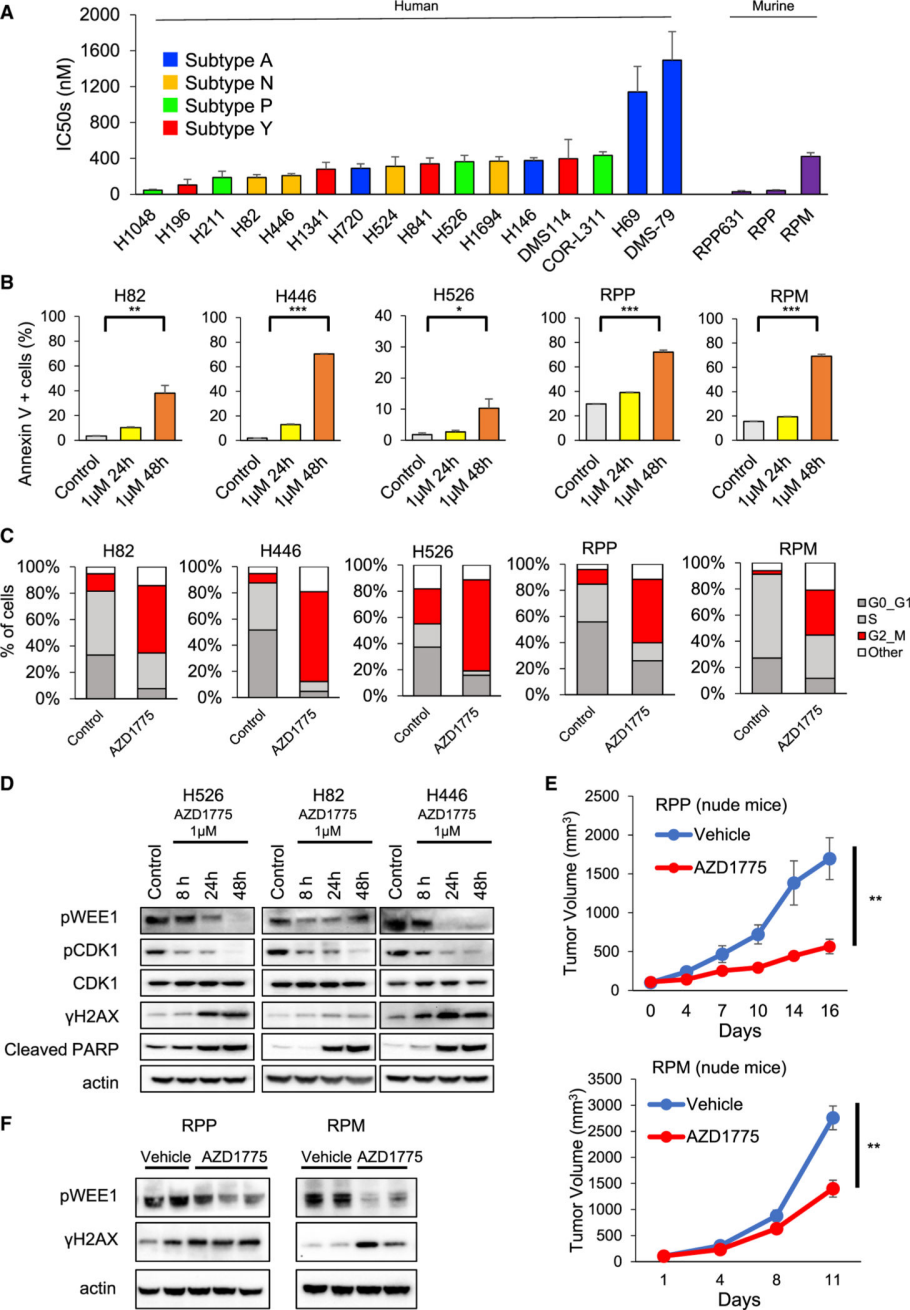
The antitumor effect of AZD1775 in SCLC (A) Cell viability IC_50_ values were determined in response to treatment with AZD1775 (0–10 μM) for 5 days in 16 human and three murine SCLC cell lines. The data shown represent the means ± SD of three individual experiments. (B) Cells were treated with 1 μM AZD1775 for 24 or 48 h. Then, the proportion of apoptotic cells was detected using annexin V-propidium iodide-based flow cytometry. Bars represent mean ± SD of triplicate. Statistical significance was determined using Student’s t test (**p < 0.01, ***p < 0.001). (C) Cells were treated with 1 μM AZD1775 for 16 h. Cell-cycle states were detected with EdU-DAPI-based flow cytometry. (D) Cells were treated with 1 μM AZD1775 for 8, 24, or 48 h. Western blots show protein expression of phospho-WEE1, phospho- and total CDK1, γH2AX, cleaved PARP, and actin (loading control) at each time indicated. (E) Tumor-growth curves of subcutaneous tumors in nude mice with conditional loss of *Trp53*, *p130*, and *Rb1* (RPP; top) and *Trp53*, *Rb1*, and *MYC*^*T58A*^ (RPM; bottom) treated with vehicle or 60 mg/kg of AZD1775 (n = 5 per group). Bars represent mean ± SE. Statistical significance was determined using Student’s t test (**p < 0.01). (F) Western blots showing phosho-WEE1, γH2AX, and actin (loading control) of RPP or RPM tumors from (E). See also Figure S1.

To confirm the sensitivity of SCLC to WEE1 inhibition, we measured the median half-maximal inhibitory concentration (IC_50_) of the WEE1 inhibitor AZD1775 in a panel of sixteen human and three murine SCLC cell lines (Figure 1A) after 5 days of treatment. SCLC cell lines displayed a range of sensitivity to AZD1775 (IC_50_s from 28.4 nM to 1.5 μM). We postulated that differential sensitivity among SCLC subtypes may drive the observed differences in sensitivity to WEE1 inhibition. However, IC_50_ values within this dataset were not significantly different across SCLC subtypes (Figure S1A). A previous clinical trial demonstrated an average maximum AZD1775 serum concentration of 1.7 μM (Do et al., 2015). The IC_50_ values for 14 of the 16 SCLC cell lines were below 1 μM. Thus, AZD1775 may have antitumor efficacy against SCLC at clinically achievable concentrations. This also established 1 μM as a relevant *in vitro* concentration for analysis of AZD1775 activity.

To initially characterize the antitumor effects of WEE1 targeting, we examined cell death using the annexin V-propidium iodide (PI) assay. Treatment with 1 μM AZD1775 for 48 h significantly increased the apoptotic fraction in human and murine SCLC cell lines relative to control (Figures 1B and S1B). WEE1 is a G2-M cell-cycle checkpoint regulator, and WEE1 inhibition should cause G2-M cell-cycle arrest (Do et al., 2013). Therefore, we next performed 5-ethynyl-2′-deoxyuridine (EdU)-4′,6-diamidino-2-phenylindole (DAPI) staining by flow cytometry to assess cell-cycle effects. Treatment with 1 μM AZD1775 induced G2-M cell-cycle arrest, consistent with previous reports (Figures 1C and S1C) (Sen et al., 2017a). Western blotting showed that phosphorylation of WEE1 and CDK1 were suppressed, while phosphorylated γH2AX (indicative of double-stranded DNA breaks [DSBs]) and cleaved PARP were increased in a time-dependent manner by treatment with 1 μM AZD1775 (Figure 1D). In summary, AZD1775 led to DNA damage and apoptosis in multiple SCLC cell lines.

Next, to elucidate the antitumor effects of AZD1775 against SCLC *in vivo*, we used cells derived from mice that were genetically engineered to spontaneously develop SCLC through conditional loss of *Trp53*, *p130*, and *Rb1* (RPP) and *Trp53*, *Rb1*, and *MYC*^*T58A*^ (RPM) (Schaffer et al., 2010; Mollaoglu et al., 2017). These genotypes closely resemble those of patients with SCLC. RPP or RPM cells were implanted in right flanks of nude mice that were treated with either vehicle or AZD1775 (60 mg/kg, 5 of 7 days, once daily [Q.D.]). Treatment with AZD1775 significantly suppressed tumor growth in both RPP and RPM models (Figure 1E), with no change in body weight (Figure S1D). Paralleling the *in vitro* data, WEE1 phosphorylation was suppressed and γH2AX expression was increased by AZD1775 (Figure 1F).

Platinum-based chemotherapy (cisplatin or carboplatin) with etoposide is an established treatment for SCLC. However, major hurdles to improving SCLC treatment include the rapid development of chemoresistance. We next investigated the effects of cisplatin or etoposide in combination with WEE1 targeting by pharmacologic inhibition (AZD1775) on cell viability in SCLC *in vitro*. In both human and murine SCLC lines, targeting WEE1 significantly enhanced cisplatin- or etoposide-mediated SCLC cell death (Figure S1E).

Thus, AZD1775 shows antitumor effects against SCLC models via G2-M cell-cycle arrest and apoptosis induction at concentrations within the pharmacologic range of AZD1775 observed in human plasma and demonstrates combinatorial cytotoxicity with both standard first-line chemotherapy drugs.

### WEE1 inhibition activates the cGAS/STING pathway and induces expression of type I interferons (IFNs) and inflammatory chemokines in SCLC models

**Figure 2.**
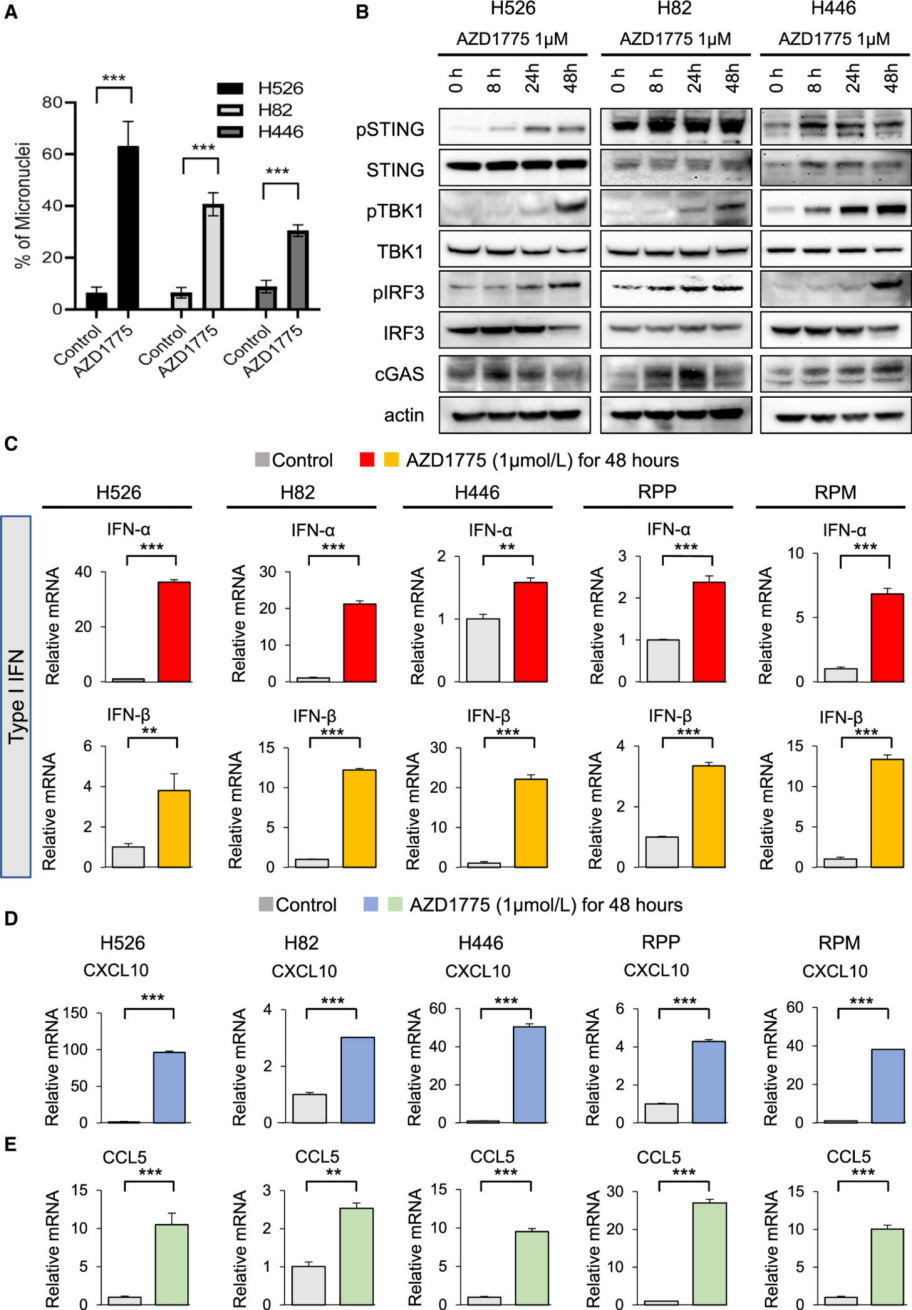
Antitumor immune response of AZD1775 is mediated via the cGAS-STING-TBK1-IRF3 pathway in SCLC (A) Quantification of cells containing micronuclei (MN) after 1 μM AZD1775 treatment for 24 h. Bars represent mean ± SD of eight areas. Statistical significance was determined using Student’s t test (***p < 0.001). (B) Western blots showing the protein expression of STING pathway, phospho (p)- and total (t)-STING, p- and t-TBK1, p- and t-IRF3, cGAS, and actin (loading control) in SCLC cells treated with 1 μM AZD1775 for 8, 24, and 48 h. (C) Quantitative mRNA expression of *IFN-α*, *IFN-β* after treatment with 1 μM AZD1775 for 48 h in SCLC cells (H526, H82, H446, RPP, and RPM). Bars represent mean ± SD of triplicate. Statistical significance was determined using Student’s t test (**p < 0.01, ***p < 0.001). (D and E) Quantitative mRNA expression of (D) *CXCL10* and (E) *CCL5* after treatment with 1 μM AZD1775 for 48 h in SCLC cells (H526, H82, H446, RPP, and RPM). Bars represent mean ± SD of triplicate. Statistical significance was determined using Student’s t test (**p < 0.01, ***p < 0.001). See also Figures S2–S4.

Based on previous studies targeting other DDR proteins including CHK1 and PARP, we hypothesized that WEE1 inhibition may activate antitumor immunity in SCLC (Sen et al., 2019a, 2019b; Zhang et al., 2020). We found that treatment with 1 μM AZD1775 for 24 h significantly increased the frequency of micronuclei in H526, H82, and H446 cells (Figure 2A and S2A). Micronuclei formation is often indicative of cytosolic DNA formation. Cytosolic DNA, as measured by cytosolic histone H3, was increased after treatment with 1 μM AZD1775, which is further indicative of DNA damage (Figure S2B).

The presence of cytosolic DNA can trigger activation of the cGAS-STING pathway, an innate antitumor immune response, in SCLC and other cancers (Sen et al., 2019a, 2019b). To investigate whether AZD1775 activated the cGAS/STING pathway in SCLC cell lines, we examined the activation of the major regulators of the pathway. Treatment with 1 μM AZD1775 led to a time-dependent activation of the cGAS/STING pathway in multiple human SCLC cell lines, as indicated by increased cGAS and phosphorylation of STING, TBK1, and IRF3 (Figure 2B).

We and others have reported that STING-pathway activation leads to induction of type I IFNs (Morel et al., 2021; Sen et al., 2019b; Takahashi et al., 2021; Zheng et al., 2020). mRNA expression of type I IFNs (*IFN-α* and *IFN-β*) significantly increased after AZD1775 treatment in human and murine cell lines (Figure 2C).

To account for potential off-target effects of AZD1775, we knocked down WEE1 using siRNA in multiple SCLC models. Genetic inhibition of WEE1 also led to activation of the STING pathway (Figure S2C) in human SCLC H82 cells, followed by a significant induction of type I IFNs (Figure S2D). The same effects were observed when we performed genetic knockdown of WEE1 in murine RPP and RPM cells (Figures S3A and S3B).

CXCL10 and CCL5 are chemokines that induce antitumor immunity in SCLC and other cancer types in response to type I IFNs (Morel et al., 2021; Sen et al., 2019b; Takahashi et al., 2021; Zheng et al., 2020). Pharmacological inhibition of WEE1 with AZD1775 (1 μM) significantly enhanced mRNA expression of *CXCL10* and *CCL5* in multiple SCLC human and murine *in vitro* models (Figures 2D and 2E).

A recent study reported that the cell-surface expression of major histocompatibility complex (MHC) class I was upregulated by WEE1 inhibition (Wu et al., 2021a). We investigated MHC class I expression in our *in vitro* models by flow cytometry. We confirm that cell-surface expression of MHC class I was increased by treatment with 1 μM AZD1775 in both human and murine SCLC cells (Figures S4A and S4B).

Together, these results suggest that inhibition of WEE1 activates the innate immune STING pathway and induces the expression of type I IFNs, and the expression of pro-tumorigenic chemokines (CXCL10, CCL5) via the induction of the STING pathway, consistent with the effects observed when targeting other DDR proteins (CHK1 and PARP).

### WEE1 inhibition enhances antitumor responses induced by anti-PD-L1 antibody in a genetically engineered mouse model (GEMM) of SCLC

**Figure 3.**
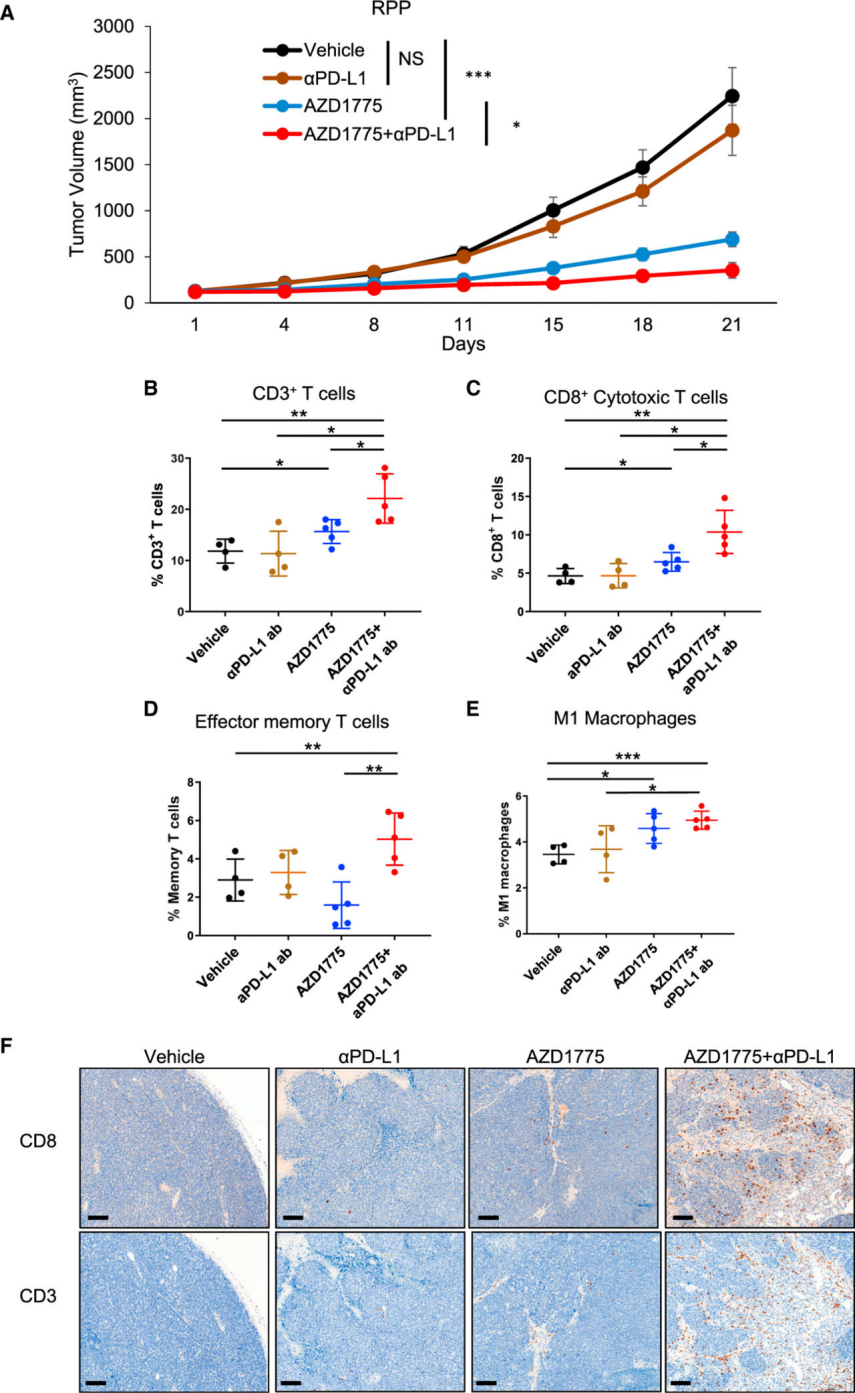
WEE1 inhibition enhances antitumor responses induced by anti-PD-L1 antibody *in vivo* in RPP tumor-bearing model (A) Tumor-growth curves of vehicle, AZD1775 alone (60 mg/kg, 5 of 7 days, Q.D.), anti-PD-L1 antibody alone (300 μg/body, once weekly), and AZD1775 plus anti-PD-L1 antibody groups in B6 129F1 mice injected with RPP cells (n = 10 per group). Bars represent mean ± SE. Statistical significance was determined using Student’s t test (*p < 0.05, ***p < 0.001). (B–E) RPP tumors were harvested at day 15 for immune profiling by flow cytometry. Cumulative data for the tumors are shown. Flow-cytometry analysis of (B) CD45^+^CD3^+^ total T cells, (C) CD45^+^CD3^+^CD8^+^ cytotoxic T cells, (D) effector memory CD8^+^ T cells: CD45^+^CD3^+^CD8^+^CD44^hi^CD62L^lo^, and (E) M1 macrophages: CD45^+^F4/80^+^GR-1^−^CD11b^+^CD68^+^iNOS^+^. Percentages were the ratio to CD45^+^ cells (n = 5 for vehicle and anti-PD-L1 group, n = 6 for AZD1775 and AZD1775 plus anti-PD-L1 group). Bars represent mean ± SD. Statistical significance was determined using Student’s t test (*p < 0.05, **p < 0.01, ***p < 0.001). (F) CD8 and CD3 immunohistochemistry was performed on sections of tumors resected on day 21 (from A). Representative images are shown. Scale bar: 100 μm. See also Figure S5.

Next, we sought to determine whether AZD1775 enhanced the antitumor immune effect of anti-PD-L1 antibody *in vivo* using the immunocompetent B6129F1 subcutaneous RPP tumor-bearing model. After randomization of tumor-bearing mice into four groups (vehicle, anti-PD-L antibody [300 μg/body, once weekly], AZD1775 [60 mg/kg, 5 of 7 days, Q.D.], and AZD1775 plus anti-PD-L1 antibody [n = 10 per group]), we treated all cohorts for 3 weeks. Treatment with anti-PD-L1 antibody alone was not efficacious in this model. In contrast, treatment with single-agent AZD1775 significantly inhibited tumor growth. Combined treatment with AZD1775 and anti-PD-L1 antibody caused significantly greater tumor suppression than either monotherapy (Figure 3A). Body weight loss was not observed in any of the groups (Figure S5A).

At day 15, a cohort of mice (vehicle [n = 4], anti-PD-L antibody [n = 4], AZD1775 [n = 5], and AZD1775 plus anti-PD-L1 antibody [n = 5]) was sacrificed, and tumors were harvested to analyze changes in tumor-infiltrating immune cells by multicolor flow cytometry (Figure S4B). Combination treatment with AZD1775 and anti-PD-L1 antibody significantly increased infiltration of CD3^+^, CD8^+^, the CD44^+^ effector/memory T cell, and M1 macrophage populations (Figures 3B–3E). Tumor-infiltrating CD4^+^ T cells tended to increase in tumors treated with AZD1775 plus anti-PD-L1 antibody, but this did not reach statistical significance (Figure S5C). Tumor-infiltrating PD-1^+^TIM3^+^ exhausted T cells (Figure S5D), M2 macrophages, and dendritic cells (data not shown) did not show any differences between groups in this model. Confirming the flow-cytometry results, Immunohistochemistry (IHC) staining indicated few changes in CD3^+^ or CD8^+^ T cells with single-agent treatment relative to vehicle; however, CD3^+^ and CD8^+^ T cell infiltration was increased in tumors treated with AZD1775 plus anti-PD-L1 antibody compared with all other groups (Figure 3F).

These results indicate that treatment with AZD1775 plus anti-PD-L1 antibody significantly increases cytotoxic T cell infiltration and enhances the antitumor effects in an *in vivo* subcutaneous model of SCLC.

### WEE1 inhibition augments antitumor immune response of PD-L1 blockade in an *MYC*-stabilized SCLC GEMM

*MYC* amplification or overexpression is one of the well-known genomic alterations in SCLC, which is observed in approximately 30% of SCLC, and RPM tumors have been previously shown to be highly aggressive and resistant to multiple therapies (Rudin et al., 2012; George et al., 2015; Chalishazar et al., 2019; Ireland et al., 2020; Mollaoglu et al., 2017). *MYC* amplification is associated with suppressed immune cell infiltrates and functional pathways (Wu et al., 2021b). Although targeting MYC in SCLC has been tested in some preclinical studies (Cargill et al., 2021), there are no defined paths for clinical translation. Furthermore, the effect of immunotherapy in the immunocompetent B6FVBF1/J subcutaneous RPM tumor-bearing model has not yet been demonstrated.

**Figure 4.**
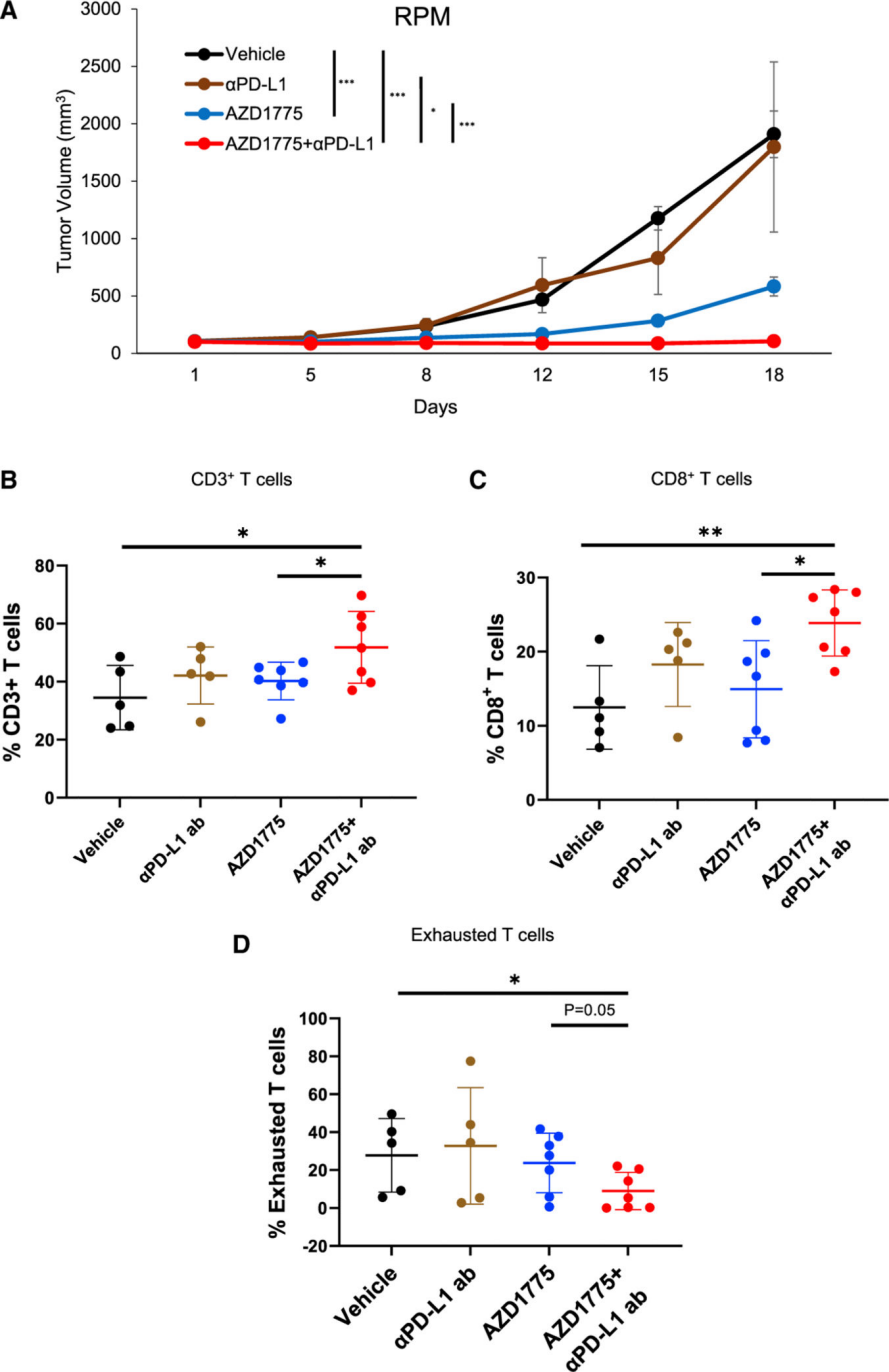
WEE1 inhibition enhances antitumor responses induced by anti-PD-L1 antibody *in vivo* in RPM tumor-bearing model (A) Tumor-growth curves of vehicle, AZD1775 alone (60 mg/kg, 5 of 7 days, Q.D.), anti-PD-L1 antibody alone (300 μg/body, once weekly), and AZD1775 plus anti-PD-L1 antibody groups in B6FVBF1/J mice injected with RPM cells (n = 5 for vehicle and anti-PD-L1 antibody groups, n = 7 for AZD1775 and AZD1775 plus anti-PD-L1 antibody groups). The data shown represent the means ± SE. p values were calculated by Student’s t test (*p < 0.05, ***p < 0.001). (B–D) RPM tumors were harvested at day 19 for immune profiling by flow cytometry. Cumulative data for the tumors are shown. Flow-cytometry analysis of (B) CD45^+^CD3^+^ total T cells, (C) CD45^+^CD3^+^CD8^+^ cytotoxic T cells, and (D) exhausted CD8^+^ T cells: CD45^+^CD3^+^CD8^+^PD-1^+^TIM-3^+^. Percentages in (B) and (C) were the ratio to CD45^+^ cells and percentages in (D) were the ratio to CD8^+^ cells (n = 5 for vehicle and anti-PD-L1 group, n = 7 for AZD1775 and AZD1775 plus anti-PD-L1 group). The data shown represent the means ± SD. p values were calculated by Student’s t test (*p < 0.05, **p < 0.01). See also Figure S6.

Therefore, we next investigated the effect of treatment with AZD1775 plus anti-PD-L1 antibody in this RPM model as an aggressive immunocompetent tumor-bearing model. We randomized the mice and treated them with vehicle (n = 5), anti-PD-L1 antibody (300 μg/body, once weekly, n = 5), AZD1775 (60 mg/kg, 5 of 7 days, Q.D., n = 7), and AZD1775 plus anti-PD-L1 antibody (n = 7). Consistent with the results of the RPP model, treatment with AZD1775 alone significantly inhibited tumor growth. The combination of WEE1 inhibition with AZD1775 and anti-PD-L1 antibody caused significant tumor regression, with tumor volumes remaining below baseline for approximately 70% of animals (Figure 4A). This is encouraging for a very aggressive tumor model that has been shown to be largely resistant to most therapies in previous studies. Body weight loss was not observed in any of the groups (Figure S6A). These results are consistent with the results that we observed in the RPP model.

Multicolor flow cytometry of tumors resected at day 19 demonstrated that combination treatment with AZD1775 and anti-PD-L1 antibody significantly increased infiltration of CD3^+^, CD8^+^ T cell populations (Figures 4B and 4C). Tumor-infiltrating PD-1^+^TIM3^+^ exhausted T cells were significantly decreased in the tumors treated with AZD1775 plus anti-PD-L1 antibody in this model (Figure 4D). CD4 helper T cells did not show any differences between group (Figure S6B).

These results indicate that treatment with AZD1775 plus anti-PD-L1 antibody significantly increases cytotoxic T cell infiltration and enhances the antitumor effects in a high-*MYC*-expressing aggressive *in vivo* subcutaneous model of SCLC.

### Gene-expression analysis demonstrated WEE1 inhibition-mediated activation of type I and II IFN pathways in SCLC mouse tumors

**Figure 5.**
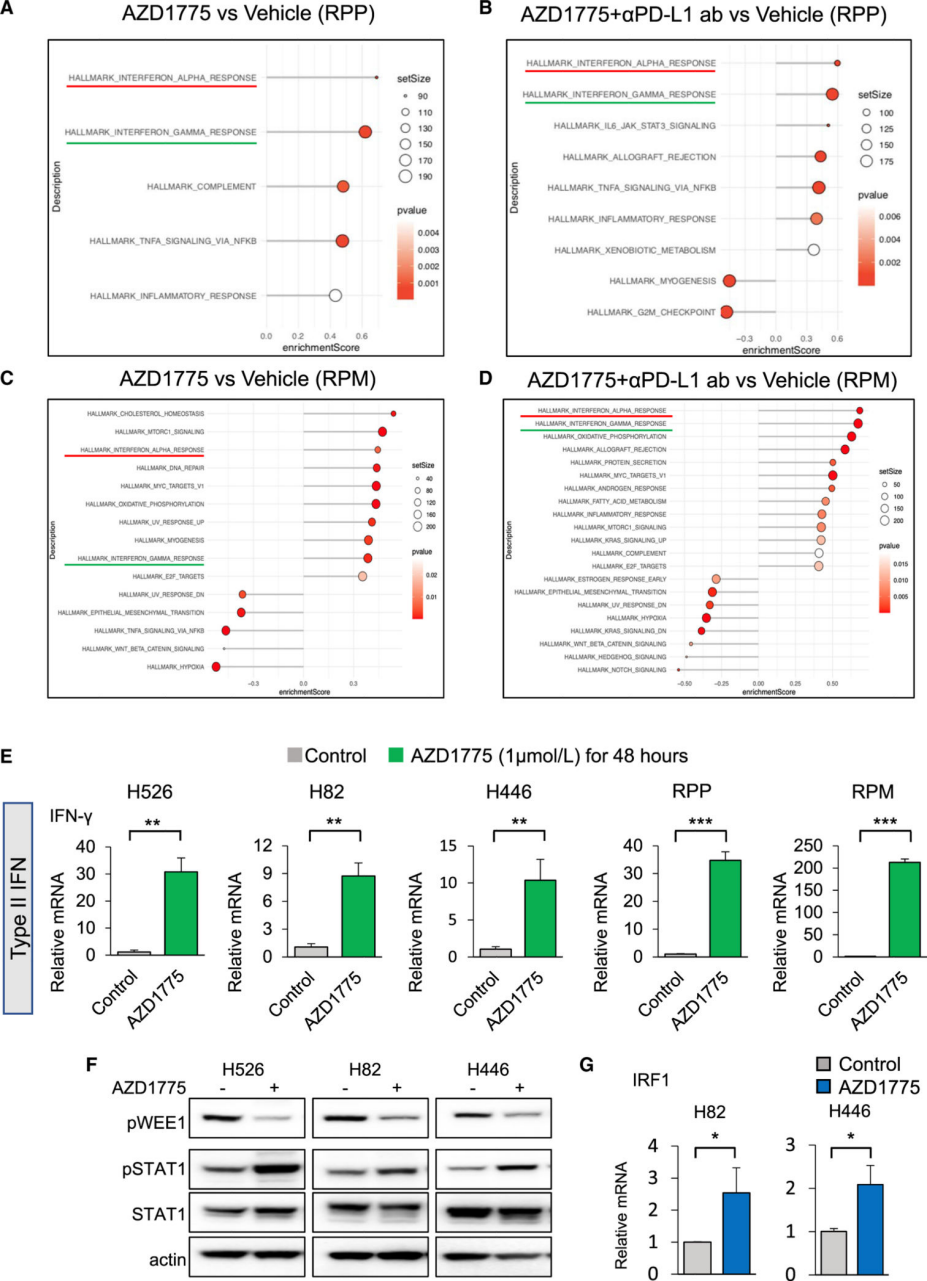
Pathway analysis demonstrates activation of type I and II interferon (IFN) pathways in SCLC mouse tumors treated with AZD1775 or AZD1775 plus anti-PD-L1 antibody, and AZD1775 mediates STAT1 pathway activation in SCLC cells (A and B) HALLMARK pathway enrichment analyses of differentially expressed genes (DEGs) from RPP tumors on the mice treated with vehicle, AZD1775, or AZD1775 plus anti-PD-L1 antibody for 21 days. (A) AZD1775 versus vehicle and (B) AZD1775 plus anti-PD-L1 antibody versus vehicle (n = 5 per group). (C and D) HALLMARK pathway enrichment analyses of DEGs from RPM tumors on the mice treated with vehicle, AZD1775, or AZD1775 plus anti-PD-L1 antibody for 18 days. (C) AZD1775 versus vehicle and (D) AZD1775 plus anti-PD-L1 antibody versus vehicle (n = 4 per group). (E) Quantitative mRNA expression of *IFN-γ* after treatment with 1 μM of AZD1775 for 48 h in SCLC cells (H526, H82, H446, RPP, and RPM cells). The data shown represent the means ± SD of triplicate. p values were calculated by Student’s t test (**p < 0.01, ***p < 0.001). (F) Western blots show expression of p-WEE1, p- and total STAT1, and actin (loading control) in SCLC cells (H526, H82, H446). Cells were treated with 1 μM AZD1775 for 24 h. (G) Quantitative mRNA expression of IRF1 after treatment with 1 μM AZD1775 for 48 h in SCLC cells (H82, H446). The data shown represent the means ± SD of triplicate. p values were calculated by Student’s t test (*p < 0.05). See also Figure S7.

We performed bulk RNA sequencing of RPP tumors and RPM tumors that were collected from each treatment group described above (Figures 3 and 4). Gene set enrichment analysis demonstrated that tumors of mice treated with AZD1775 alone or in combination with anti-PD-L1 antibody exhibited significant enrichment of IFN-α/β pathways compared with controls in both RPP and RPM tumors (Figures 5A–5D). IFN-α/β pathways in RPM tumors (but not RPP tumors) treated with AZD1775 in combination with anti-PD-L1 antibody were significantly enriched relative to AZD1775 alone (Figure S6C). This is in agreement with the similar induction of these genes we observed in SCLC cell lines.

Interestingly, in addition to the type I IFN-α pathway, we also observed the IFN-γ pathway to be one of the top significantly enriched pathways in the tumors treated with AZD1775 alone or in combination with anti-PD-L1 antibody (Figures 5A–5D). Previous reports in other cancers have highlighted the effect of DSBs on type II IFN (IFN-γ) induction (Higuchi et al., 2015; Huang et al., 2015). However, there are no reports of DDR-mediated induction of type II IFNs in SCLC, and the role of IFN-γ in SCLC is unknown. To confirm DDR-mediated IFN-γ induction, we next investigated IFN-γ expression in SCLC cell lines treated with AZD1775. Consistent with RNA sequencing, treatment with 1 μM AZD1775 significantly increased *IFN-γ* mRNA expression in human and murine SCLC cell lines (Figure 5E). Genetic inhibition of WEE1 also led to *IFN-γ* mRNA expression in human and murine SCLC cell lines (Figure S7A).

Therefore, our data indicate that WEE1 inhibition leads to concomitant induction of type I (IFN-α/β) and II (IFN-γ) IFNs in SCLC *in vitro* and *in vivo* models.

### STAT1 pathway regulates WEE1 inhibition-mediated IFN-γ induction and PD-L1 expression in SCLC

Previous reports have shown that the IFN-γ can induce the JAK-STAT-IRF1 pathway in the tumor microenvironment (Garcia-Diaz et al., 2017). We therefore explored the influence of WEE1 inhibition on JAK-STAT signaling. Relative to control, treatment with 1 μM AZD1775 appreciably increased STAT1 phosphorylation in three human SCLC cell lines (Figure 5F). Furthermore, 1 μM AZD1775 significantly increased mRNA expression of IRF1, which is a transcription factor that is induced by the accumulation of phosphorylated STAT1 dimers (Figure 5G) (Michalska et al., 2018).

Previous studies have shown that IFN-γ induces PD-L1 expression in several malignancies (Gocher et al., 2021), but this mechanism has not been previously explored in SCLC. We have previously shown that CHK1 and PARP targeting induces protein and surface expression of PD-L1 in SCLC (Sen et al., 2019b). To explore the effect of WEE1 inhibition on PD-L1 expression, we treated a panel of human SCLC cell lines (n = 8), as well as RPP and RPM murine cell lines, with AZD1775 (1 μM) for 72 h and analyzed PD-L1 surface expression by flow cytometry. Consistent with observations in CHK1 and PARP, the mean fluorescence intensity (MFI) of PD-L1 on the cell surface increased following treatment with 1 μM AZD1775 (Figures S7B and S7C). To confirm that PD-L1 upregulation is specifically due to inhibition of WEE1 and not an off-target effect of the inhibitor, we knocked down WEE1 with small interfering RNA (siRNA) and examined expression by flow cytometry, and a similar increase in PD-L1 MFI was observed (Figure S7D). The IHC staining of the tumors in the RPP mouse model (Figure 3) also indicated AZD1775-treated tumors to have a significantly higher number of PD-L1^+^ cells per field compared with the vehicle group (Figures S7E and S7F). Flow cytometry indicated that exogeneous recombinant IFN-γ induced PD-L1 expression (increased MFI) in SCLC cells (Figures S7G and S7H).

**Figure 6.**
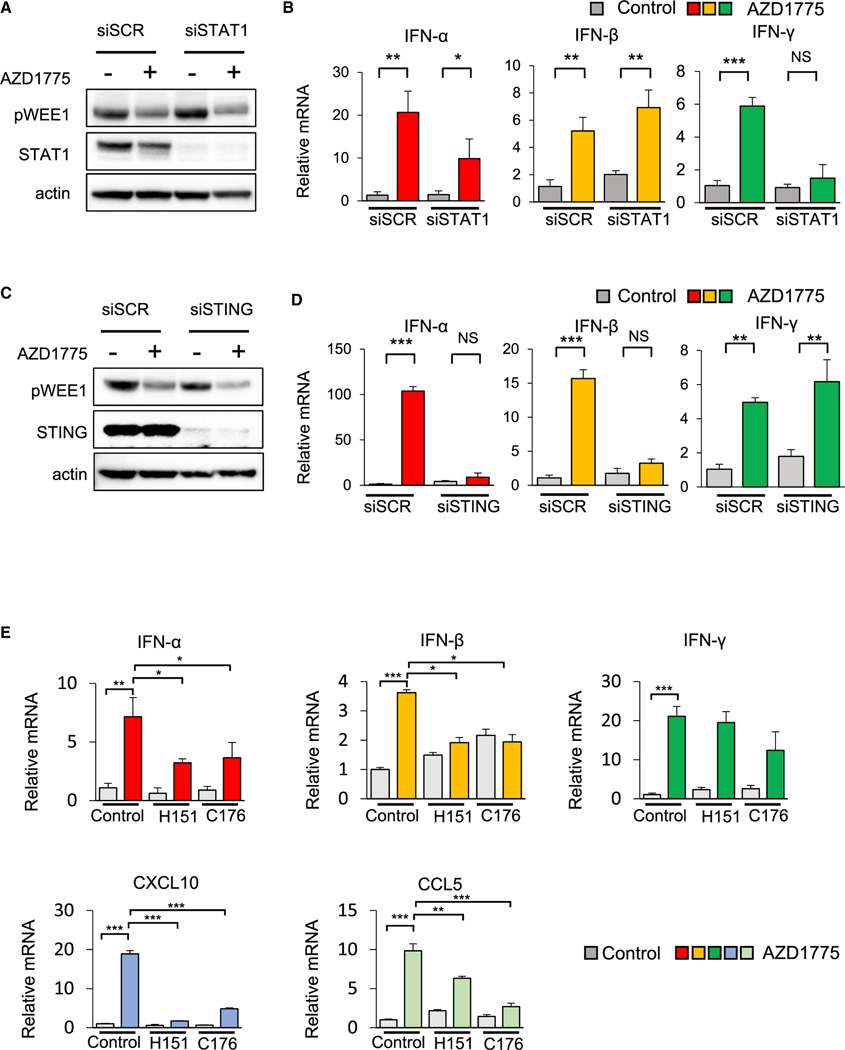
STING and STAT1 mediate induction of type I and type II interferons following WEE1 inhibition (A and B) Knockdown of STAT1 (or siRNA control [siSCR]) by siRNA followed by treatment with 1 μM AZD1775 for 48 h in H82 cells. (A) Western blots show expression of p-WEE1, total STAT1, and actin (loading control). (B) Quantitative mRNA expression of *IFN-α*, *IFN-β*, and *IFN-γ*. (C and D) Knockdown of STING (or siSCR) by siRNA followed by treatment with 1 μM AZD1775 for 48 h in H82 cells. The data shown represent the means ± SD of triplicate. p values were calculated by Student’s t test (*p < 0.05, **p < 0.01, ***p < 0.001). (C) Western blots showing expression of p-WEE1, total STING, and actin (loading control). (D) Quantitative mRNA expression of *IFN-α*, *IFN-β*, and *IFN-γ*. The data shown represent the means ± SD of triplicate. p values were calculated by Student’s t test (**p < 0.01, ***p < 0.001). (E) Quantitative mRNA expression of *IFN-α*, *IFN-β*, *IFN-γ*, *CXCL10*, and *CCL5* after treatment with 500 nM H151 or 1 μM C176 and 1 μM AZD1775 for 48 h in H526 cells. The data shown represent the means ± SD of triplicate. p values were calculated by Student’s t test (*p < 0.05, **p < 0.01, ***p < 0.001). See also Figure S8.

To further dissect the influence of STING and STAT1 pathways on IFN-α/β and IFN-γ expression, respectively, we next performed siRNA-mediated knockdown of *STAT1* or *STING* in SCLC cells. Cells with *STING* or *STAT1* knockdown were then treated with AZD1775. Knockdown of *STAT1* (confirmed by western blot; Figure 6A) did not influence AZD1775-mediated upregulation of *IFN-α* and *IFN-β* but prevented AZD1775-induced upregulation of *IFN-γ* (Figure 6B). Knockdown of *STAT1* also suppressed AZD1775-mediated PD-L1 expression (Figure S8A). In contrast, knockdown of *STING* (confirmed by western blot; Figure 6C) did not influence AZD1775-mediated upregulation of *IFN-γ* and instead prevented AZD1775-induced upregulation of *IFN-α* and *IFN-β* (Figure 6D). Consistent with these findings, STING inhibitor H151 or C176 blocked AZD1775-induced upregulation of STING phosphorylation, *IFN-α*, *IFN-β*, *CXCL10*, and *CCL5* but did not inhibit upregulation of STAT1 phosphorylation and *IFN-γ* (Figures 6E, S8B, and S7C).

Taken together, we demonstrate that WEE1 inhibition activates type I IFNs (IFN-α and IFN-β) via the STING pathway and type II IFN (IFN-γ) via the STAT1 pathway. In our SCLC models, STAT1 activation mediated by WEE1 inhibition also contributed to increased PD-L1 expression. Thus, our results indicate multi-modal immune activation with WEE1 targeting in SCLC. WEE1 inhibition activated the cGAS/STING/TBK1/IRF3 pathway followed by increased IFN-α, IFN-β, CXCL10, and CCL5, which ultimately led to CD8^+^ T cell recruitment in multiple SCLC *in vivo* models. In addition, WEE1 inhibition led to STAT1-pathway activation, which in turn induced IFN-γ and PD-L1 expression. This multi-modal immune activation significantly augmented the anti- tumor immune response of anti-PD-L1 immunotherapy in SCLC.

## DISCUSSION

Drugs targeting DDR proteins, including WEE1, are under preclinical and clinical development either as single agents or in combination with ICB for patients with multiple cancers (Do et al., 2015; Färkkilä et al., 2020; Fumet et al., 2020) (ClinicalTrials.gov: NCT04633902, NCT04782089, NCT04276376, and NCT04483544). Here, we report the tumor-intrinsic and previously unexplored roles of WEE1 pathway targeting in regulating the antitumor immune response in multiple SCLC models. We show that inhibition of WEE1 potentiates the antitumor immune response of anti-PD-L1 antibody by concomitant activation of the cGAS/STING and STAT1 pathways, which ultimately enhances expression of the type I and type II IFN genes, respectively. The type I IFNs (IFN-α/β) increase expression of downstream chemokines such as CXCL10 and CCL5, leading to induced recruitment of CD8+ cytotoxic T lymphocytes. The type II IFN (IFN-γ) induces the expression of PD-L1, which may present an immunological therapeutic opportunity. When combined with anti-PD-L1 antibody, WEE1 targeting demonstrates a significant antitumor effect in multiple SCLC models, suggesting that this combination may be valuable clinically to overcome primary and adaptive resistance to ICB in SCLC.

The genomic profile of SCLC includes nearly ubiquitous genetic loss of *TP53* and *RB1* and thus the loss of G1/S cell-cycle checkpoint control in response to DNA damage (Sen et al., 2018; Rudin et al., 2012; George et al., 2015; Alexandrov et al., 2013). Additionally, about 15% of SCLC tumors have *MYC* amplification (Sos et al., 2012), thereby providing additional oncogenic stresses during the tumor cell cycle. As a result, the rapidly dividing SCLC tumor cells are under substantial replication stress and are heavily reliant on G2/M cell-cycle checkpoint proteins, like WEE1, to maintain survival (Sen et al., 2018). Our findings suggest that this reliance on WEE1 represents a tumor-selective vulnerability in SCLC. WEE1 inhibition in SCLC leads to replicative dysfunction, resulting in aberrant trafficking of DNA fragments into the cytoplasm and activation of the inflammatory cascade described above.

We and others have shown that DDR components (CHK1, PARP) are overexpressed in SCLC and that inhibitors of these targets are broadly active in preclinical models of SCLC (Sen et al., 2019a, 2019b). Our results demonstrate that pharmacological or genetic inhibition of WEE1 is sufficient to induce cell death via apoptosis in SCLC, across all subtypes, in agreement with prior observations (Sen et al., 2017a). The current study shows the remarkable effect of single-agent WEE1 targeting in syngeneic *in vivo* models of SCLC. Most notably, the current study provides mechanistic insight into both tumor-intrinsic and immunologic effects of WEE1 targeting in SCLC.

The benefit of adding ICB to chemotherapy as a first-line standard treatment for patients with SCLC is limited: durable responses remain rare, and more consistently durable therapeutic approaches are needed. PD-L1 expression on SCLC is low compared with the range seen in non-SCLCs (NSCLCs) and other solid tumors, and an association between PD-L1 expression and the effect of immunotherapy has not yet been established in SCLC (Taniguchi et al., 2020). In contrast, T cell infiltration in SCLC tumors appears to be a prognostic marker associated with improved survival (Eerola et al., 2000; Poirier et al., 2020). Thus, the therapeutic combinations that enhance the effects of ICB may provide benefit via activation of T cells. Patients with cancers harboring innate defects in DDR genes have increased intratumoral CD8^+^ T cell infiltration and improved response to ICB (Le et al., 2015; Sun et al., 2021; Teo et al., 2018). However, the intertwined relationship between immunotherapies and DDR pathways is complex and appears to be DDR-protein specific. The synergy between DDR inhibition and immune activation in some but not all cancer models is clearly multifactorial and context specific. The current study adds to our prior analyses of CHK1 and PARP inhibition on the immune microenvironment of SCLC (Sen et al., 2019a, 2019b) and nominates WEE1 as a therapeutic target with immune-modifying activity.

Previous studies demonstrated that targeting the DDR pathway with PARP or CHK1 inhibitors led to an accumulation of cytosolic DNA, which activated the cGAS/STING/TBK1/IRF3 pathway and, in turn, directly promoted expression of type I IFNs in SCLC (Sen et al., 2019a, 2019b). Consistent with those prior observations, here, we demonstrated that WEE1 inhibition *in vitro* increased DNA damage, micronuclei formation, and subsequent cytosolic DNA accumulation. As with PARP and CHK1 inhibitors, we observed activation of the cGAS/STING innate immune pathway and TBK1, which led to IRF3 phosphorylation and induction of type I IFNs. We also observed increased expression of CXCL10 and CCL5 after WEE1 inhibitor treatment, which was abrogated after STING inhibition. Previous reports suggested that type I IFNs are necessary for T cell-mediated responses against tumor antigens (Woo et al., 2015). WEE1 inhibition enhanced expression of both type I and type II IFN genes. Induction of type II IFN following DDR inhibition and its mechanism have not been previously explored in SCLC. Our results demonstrate that type II IFN could indirectly regulate PD-L1 levels in these tumors.

In summary, WEE1 inhibition induces G2/M cell-cycle arrest, DNA damage, and cytosolic DNA accumulation in SCLC models. WEE1 inhibition further concomitantly activates cGAS/STING, resulting in induction of type I IFNs, CXCL10, and CCL5. Activation of the STING-mediated pathway is responsible for chemokine production in response to DNA damage, thereby resulting in increased immunogenicity of the otherwise immunosuppressed tumors. WEE1 inhibition also activates the STAT1 pathway leading to IFN-γ and PD-L1 expression, which may partly abrogate the antitumor immune effects of WEE1 inhibitor. Consistent with these models, combining WEE1 inhibition with PD-L1 blockade markedly suppressed tumor growth and increased CD8^+^ T cells in the tumor. Our findings demonstrated a pivotal role of WEE1 inhibition in augmenting the antitumor immune response of ICB in SCLC. Given the increasing importance of immunotherapy for the management of patients with SCLC and that WEE1 inhibitors are already in clinical trials, combining a WEE1 inhibitor with anti-PD-L1 blockade may offer a particularly attractive strategy for the treatment of SCLC.

### Limitations of the study

Our results highlight the antitumor immune effects of AZD1775 either alone or in combination with PD-L1 blockade in preclinical models of SCLC. Whether WEE1 inhibition alone or in combination with chemoimmunotherapy suppresses the growth of tumors in patients with SCLC, and the immunological effects of this combination treatment *in vivo*, should be further investigated.

An outstanding question concerns whether WEE1 inhibition augments the antitumor immune response of chemoimmunotherapy in platinum-sensitive and -resistant models. Our data show that treatment with AZD1775 plus cisplatin or etoposide further decreased viability in SCLC cell lines. However, in both mouse and human SCLC models (treatment naive or relapsed), the evaluation whether there is benefit from combining WEE1 inhibition with chemoimmunotherapy on tumor growth and on related to immune subsets will be a focus of future work. To fully evaluate the effect of AZD1775 plus anti-PD-L1 antibody with standard chemotherapy on the immune repertoire, additional syngeneic mouse models deficient in different components of the immune system would be highly informative.

Another outstanding question is whether WEE1 inhibition changes the mutational repertoire in clinical samples and how that might influence the immune microenvironment. The rapid onset of tumor reduction following treatment with AZD1775 in immunocompetent SCLC mouse models in our study suggests that activation of the innate immune response—rather than change in neoantigens—is central to the antitumor response. However, future studies to monitor the mutational profile, neoantigen landscape, microenvironment, and systemic immunologic effects of WEE1 inhibition in preclinical models and in patients with SCLC will be valuable to further inform clinical translational strategies for optimal deployment of WEE1 inhibitors with immunotherapy in this setting.

## STAR★METHODS

### RESOURCE AVAILABILITY

#### Lead contact

Further information and requests for resources and reagents should be directed to and will be fulfilled by the lead contact, Triparna Sen (sent@mskcc.org).

#### Materials availability

This study did not generate new unique reagents.

#### Data and code availability

- RNA seq data have been deposited at ArrayExpress and are publicly available as of the date of publication. Accession numbers are listed in the key resources table.
- This paper does not report original code.
- Any additional information required to reanalyze the data reported in this paper is available from the lead contact upon request.

### EXPERIMENTAL MODEL AND SUBJECT DETAILS

#### Cell lines and cell cultures

SCLC human-derived cell lines were obtained from the American Type Culture Collection (ATCC) and European Collection of Authenticated Cell Cultures (ECACC) (Key resources table). The GEMM-derived SCLC cell lines derived from a triple-knockout model of SCLC, Trp53−/−, p130−/−, Rb1−/− (RPP) were kindly provided by Dr. Julien Sage, Stanford University, California. RPP631 cells were kindly provided by Dr. Matthew G Oser, Dana-Farber Cancer Institute, Boston, and Rb1−/−, Trp53−/−, MYC^T58A^ (RPM) cells were kindly provided by Trudy G. Oliver, University of Utah, Utah.

All cell lines were maintained in Roswell Park Memorial Institute (RPMI) media supplemented with 10% fetal bovine serum, penicillin (100 U/mL), and streptomycin (50 g/mL) and incubated at 37^◦^C with 5% CO_2_. RPP631 cells were maintained as above with addition of 0.005 mg/mL insulin, 0.01 mg/mL transferrin, 30nM sodium selenite, 10 nM hydrocortisone, and 10 nM beta estradiol.

All cell lines were tested and authenticated by short tandem repeat profiling (DNA fingerprinting) and routinely tested for mycoplasma species before any experiments were performed.

#### Mouse models

All animal experiments were approved by the Memorial Sloan Kettering Cancer Center (MSKCC) Animal Care and Use Committee. Female nude mice (6 weeks old) were obtained from ENVIGO, female B6 129F1 (6 weeks old) were obtained from TACONIC, female B6 FVBF1/J (6 weeks) and female NSG mice (6 weeks) were obtained from JACKSON LABORATORY, and housed in accredited facilities under pathogen-free conditions. Additional information on experimental methods in next section.

### METHOD DETAILS

#### Chemical compounds

AZD1775, H151 and C176 were purchased from Selleck chemical (Houston, TX). Anti-PD-L1 antibodiy (clone 10F.9G2) and IgG isotype control were purchased from BioXcell (West Lebanon, NH). Cisplatin and etoposide were purchased from Accord Healthcare (Durham, NC). Recombinant IFN-γ was purchased from Biolegend (San Diego, CA).

#### Cell viability assay

1,500 to 2,000 SCLC cells were plated in 96-well plates and treated with dimethyl sulfoxide (vehicle) or AZD1775 for five days for the calcuration of half-maximal inhibitory concentrations (IC50s), or treated with AZD1775, cisplatin and/or etoposide for 72 h. Cell viability assay was performed using with CellTiter-Glo luminescent cell viability assay (Promega, Madison, WI) in accordance with the manufacturer’s instructions. IC50s were estimated using GraphPad Prism Ver. 9.0 (GraphPad Software, Inc., San Diego, CA, USA).

#### Western blotting

Protein extractions were performed as previously described (Wohlhieter et al., 2020). Extraction of separate cytoplasmic and nuclear protein fractions were performed using NE-PER™ Nuclear and Cytoplasmic Extraction Reagents (Thermo Fisher) according to the manufacturer’s protocol. Briefly, 30–50 μg of protein were mixed with NuPAGE LDS sample buffer (Invitrogen), loaded in a Bis-Tris Gel (NuPAGE, Invitrogen) and resolved. Electrophoresed protein samples were transferred with Trans-Blot Turbo RTA Mini LF PVDF Transfer Kit (Bio-Rad, Alfred Nobel Drive Hercules, CA) for chemiluminescent detection. After incubating with Pierce Starting Block (PBS) Blocking Buffer (Thermo Fisher) at room temperature for 30 min, membranes were incubated in the primary antibodies (1:1000) overnight (the antibodies’ information are in Key resources table). Secondary anti-rabbit, horseradish peroxidase-linked antibodies were purchased from CST (#7074) and detected using iBright Western Blot Imaging Systems (Thermo Fisher).

#### Real-time PCR

RNA extractions were performed using the RNeasy Plus Mini Kit (Qiagen, Germantown, MD) according to the manufacturer’s protocol. cDNA was synthesized from RNA by reverse transcription PCR using Superscript IV VILO Master Mix (Fisher Scientific) and real-time PCR was performed using TaqMan Fast Advanced Master Mix (Invitrogen) according to the manufacturer’s protocol. Triplicate PCR reactions were run on StepOnePlus Real-Time PCR System (Applied Biosystems) and 2^−ΔΔ^ method were used for comparative Ct. *GAPDH* was used as the reference gene for these calculations. The probes which were used in this study are listed in the Table S1.

#### Flow cytometry

For *in vitro* assay, the cells were incubated with anti-CD16/32 monoclonal antibody (for murine cells) or TruFCX (for human cells) to block nonspecific binding, and then stained (30 min) with aintibody in appropriate dilutions. Dead cells were excluded on the basis of 4′,6-diamidino-2-phenylindole (DAPI, 1μg/mL). For analyzing the mouse tumors, single-cell suspensions were prepared using with the gentleMACS dissociator (Miltenyi Biotec, Auburn, CA) according to the manufacturer’s protocol. Single-cell suspensions were stained with anti-CD16/32 monoclonal antibody and fluorochrome-conjugated antibodies for 30 min then fixed with 1% Paraformal- dehyde overnight. Intracellular Staining Permeabilization Wash Buffer (Biolegend, San Diego, CA) were used for intracellular staining. Antibodies for flow cytometry are listed in the Table S2. Data were obtained on a Cytek Aurora (Cytek, Bethesda, MD) flow cytometer and analyzed with FlowJo software (version 10.6, BD Biosciences (Franklin Lakes, NJ)).

#### Annexin V-propidium iodide (PI) assay

2–4×10^5^ cells were plated in six-well plates and treated with or without 1 μM AZD1775 for 24 or 48 h. Cells were harvested and stained with annexin V and propidium iodide using the FITC annexin V Apoptosis Detection Kit I (BD Biosciences) according to the manufacturer’s protocol. Data were obtained on a Cytek Aurora (Cytek) flow cytometer and analyzed with FlowJo software (version 10.6, BD Biosciences).

#### EdU-DAPI based flow cytometry

2–4×10^5^ cells were plated in six-well plate and treated with or without 1 μM AZD1775 for 16 h. Before harvesting, cells were incubated with 10 μM EdU for two hours. The cells were harvested stained with Click-iT EdU Alexa Fluor 488 Flow Cytometry Assay Kit (Invitrogen) according to the manufacturer’s protocol. Data were obtained on a Cytek Aurora (Cytek) flow cytometer and analyzed with FlowJo software (version 10.6, BD Biosciences).

#### Micronuclei assay

Cells were treated with or without AZD1775 (1μM). Cytochalasin B (3 μg/mL) was added at the 20th hour to each culture and incubated at 37^◦^C. After 24hrs, the cells were centrifuged at 1000 rpm for 5 min. The supernatant was removed, and the pellet was treated with weak hypotonic solution (0.075 M KCl/0.9% Saline, 1:9) and incubated at 37^◦^C for 5 min. After this, the cells were centrifuged and the pellets were fixed in fresh fixative (methanol:acetic acid, 3:1). Cells were dropped onto glass slides prepared and stained with ProLong Gold Antifade Mountant with DAPI (CST) for scoring. The slides were scanned with a 20x/0.8NA plan-apochromat objective in a 3DHistech Pannoramic 250 Flash Scanner (3DHISTECH, Öv u. 3., Hungary). The scanned images were evaluated with Case Viewer (3DHISTECH) in the setting of 20x in Case Viewer. At least 10 areas following the standard specifications, were scored for each slide.

#### Transfection of siRNAs

Silencer Select siRNAs for WEE1 (s21 and s23 for human, s76041 and s76042 for murine) and STING (s50644) were purchased from Invitrogen (Carlsbad, CA) and STAT1 (L-003543–00–0005) were purchased from Horizon Discovery (Lafayette, CO). SiRNAs were transfected into cells using lipofectamine RNAi-MAX (Invitrogen) in accordance with the manufacturer’s protocol. In all experiments, Silencer Select siRNA for Negative Control no.1 (Invitrogen) was used as the scrambled control. Knockdown was confirmed by western blotting analysis. Each sample was tested in triplicate with three independent experiments being performed.

#### Nude mouse model

Suspensions of 2×10^6^ cells were injected subcutaneously in a 1:1 mixture of phosphate-buffered saline (PBS) and Matrigel (#CB40234, Fisher) into the flanks of 6-week-old female nude mice obtained from ENVIGO (Boyertown, PA). Once the mean tumor volume reached approximately 100 mm^3^, mice were randomized and treated with either vehicle (0.5% methylcellulose) or AZD1775 (60 mg/kg, 5 of 7 days, Q.D.; *n* = 5/group). Tumors were measured twice a week using calipers and their volumes were calculated as width^2^ x length x 0.5. Body weights were monitored twice a week. The Student t-test was used to determine statistical significance between two group groups.

#### Immunocompetent mice model

Cell suspensions (2×10^6^ RPP cells) were injected subcutaneously in a 1:1 mixture of PBS and Matrigel (#CB40234, Fisher) into the flanks of 6-week-old female B6129F1 mice obtained from TACONIC. For the RPM-tumor model, suspensions of 3×10^6^ RPM cells were injected into the flanks of 6-week-old female NSG mice (The Jackson Laboratory, Bar Harbor, ME). Two weeks later, tumors were harvested and processed into single-cell suspensions using with the gentleMACS dissociator (Miltenyi Biotec). The single-cell suspensions (3×10^6^ RPM cells) were injected into 6-week-old female B6FVBF1/J mice (The Jackson Laboratory). Once the mean tumor volume reached approximately 100 mm^3^, mice were randomized and treated with either vehicle (0.5% Methylcellulose), anti-PD-L1 antibody (300mg/body, once weekly, Intraperitoneal injection), AZD1775 (60 mg/kg, 5 of 7 days, Q.D., oral gavage), or AZD1775 and anti-PD-L1 antibody. Tumors were measured twice a week using calipers and their volumes were calculated as width^2^ x length x 0.5. Body weights were monitored twice a week. The Student t-test was used to determine statistical significance between two groups.

#### RNA sequencing

Fresh frozen tumor samples were shipped to GENEWIZ for RNA isolation, library preparation, and RNA sequencing. Total RNA was extracted using Qiagen RNeasy Plus Universal mini kit following manufacturer’s instructions (Qiagen, Hilden, Germany). Extracted RNA samples were quantified using Qubit 2.0 Fluorometer (Life Technologies, Carlsbad, CA, USA) and RNA integrity was checked using Agilent TapeStation 4200 (Agilent Technologies, Palo Alto, CA, USA). RNA sequencing libraries were prepared using the NEBNext Ultra II RNA Library Prep Kit for Illumina following manufacturer’s instructions (NEB, Ipswich, MA, USA). Briefly, mRNAs were first enriched with Oligo(dT) beads. Enriched mRNAs were fragmented for 15 min at 94^◦^C. First strand and second strand cDNAs were subsequently synthesized. cDNA fragments were end repaired and adenylated at 3′ends, and universal adapters were ligated to cDNA fragments, followed by index addition and library enrichment by limited-cycle PCR. The sequencing libraries were validated on the Agilent TapeStation (Agilent Technologies, Palo Alto, CA, USA), and quantified by using Qubit 2.0 Fluorometer (Invitrogen, Carlsbad, CA) as well as by quantitative PCR (KAPA Biosystems, Wilmington, MA, USA). The sequencing libraries were clustered on 1 flowcell lane. After clustering, the flowcell was loaded on the Illumina HiSeq instrument (4000 or equivalent) according to manufacturer’s instructions. The samples were sequenced using a 2 × 150bp Paired End (PE) configuration. Image analysis and base calling were conducted by the HiSeq Control Software (HCS). Raw sequence data (.bcl files) generated from Illumina HiSeq was converted into fastq files and de-multiplexed using Illumina’s bcl2fastq 2.17 software. One mismatch was allowed for index sequence identification.

#### RNA-seq analysis

We quantified RPP RNA-seq reads with Kallisto v.0.45.0 (Pimentel et al., 2017) to obtain transcript counts and abundances. Kallisto was run with 100 bootstrap samples, sequence based bias correction, and in strand specific mode, which processed only the fragments where the first read in a pair is pseudoaligned to the reverse strand of a transcript. RNA-seq of RPM was quantified with Salmon v1.1.0 (Patro et al., 2017). Salmon was run on raw reads mapped to 25 mer indexed mm10 genome. In addition to default settings, mapping validation (–validatemappings), bootstrapping with 30 re-samplings (–numBootstraps), sequence specific biases correction (–seqBias), coverage biases correction (–posBias) and GC biases correction (–gcBias) were enabled. Differential gene expression analysis, principle component analysis, and transcript per million (TPM) normalization by size factors, were done from Salmon output files using Sleuth v0.30.0 run in gene mode (Pimentel et al., 2017). The transcript to gene map was based on Ensembl 92 (Zerbino et al., 2018). Differentially expressed genes were identified using the Wald test. Genes were marked as significant if the False Discovery Rates, q, calculated using the Benjamini-Hochberg menthod, was less than 0.05, and beta (Sleuth-based estimation of log2 fold change) > 0.58, which approximately correlated to a log2 fold change of 1.5 in our data.

Gene set enrichment analysis (GSEA) (Subramanian et al., 2005) was performed on full sets of differential gene expression data across the previously mentioned comparisons. Genes were ranked on p value scores computed as -log10(p value)*(sign of beta). Gene set annotations were Hallmark genes taken from Molecular Signatures Database (MSigDB v7.0.1) (Subramanian et al., 2005) (Liberzon et al., 2011). The significance level of enrichment was evaluated using permutation test and the p value was adjusted by Benjamini-Hochberg procedure. Any enriched gene sets with adjusted p value ≤0.05 were regarded as significant. This analysis was conducted using the R package ClusterProfiler v3.18.1 (Yu et al., 2012).

#### Histological analyses of tumors

Tissue were fixed in 10% neutral buffered formalin, processed in ethanol and xylene, and infiltrated with paraffin in a Leica ASP6025 tissue processor. Paraffin blocks were sectioned at 5 μm thickness and immunohistochemistry was performed with anti-CD3, anti-CD8a, anti-PD-L1 antibodies. Immunohistochemistry was carried out on a Leica Bond RX automated stainer using Bond reagents (Leica Biosystems, Buffalo Grove, IL), including a polymer detection system (DS9800, Novocastra Bond Polymer Refine Detection, Leica Biosystems). The chromogen was 3,3 diaminobenzidine tetrachloride (DAB), and sections were counterstained with hematoxylin. Details for each marker are shown in the Table S3. The slides were scanned with a 20x/0.8NA plan-apochromat objective in a 3DHistech Pannoramic 250 Flash Scanner (3DHISTECH). The scanned images were evaluated with Case Viewer (3DHISTECH). The number of PD-L1 positive cells were counted in the setting of 20x in Case Viewer from five areas of five tumors in each group.

### QUANTIFICATION AND STATISTICAL ANALYSIS

Data from RT-PCR, flow cytometry, micronuclei assay, and histological analysis were expressed as means ± standard deviation (SD) and tumor progression in animal studies as means ± standard error (SE), respectively. The statistical significance of differences was analyzed using with GraphPad Prism Ver. 9.0 with p value less than 0.05 considered statistically significant (ns > 0.05, *p < 0.05, **p < 0.01, ***p < 0.001).

## Supplementary Material

1

## Figures and Tables

**Table:** KEY RESOURCES TABLE

REAGENT or RESOURCE	SOURCE	IDENTIFIER
Antibodies		

Phospho-Wee1 (Ser642)	Cell Signaling Technology	Cell Signaling Technology Cat# 4910, RRID:AB_2215870
STING (D2P2F)	Cell Signaling Technology	Cell Signaling Technology Cat# 13647, RRID:AB_2732796
Phospho-STING (Ser366)	Cell Signaling Technology	Cell Signaling Technology Cat# 19781, RRID:AB_2737062
TBK1	Cell Signaling Technology	Cell Signaling Technology Cat# 3504, RRID:AB_2255663
phospho-TBK1(S172)	Cell Signaling Technology	Cell Signaling Technology Cat# 5483, RRID:AB_10693472
Stat1	Cell Signaling Technology	Cell Signaling Technology Cat# 14994, RRID:AB_2737027
Phospho-Stat1 (Ser727)	Cell Signaling Technology	Cell Signaling Technology Cat# 8826, RRID:AB_2773718
IRF-3	Cell Signaling Technology	Cell Signaling Technology Cat# 11904, RRID:AB_2722521
Phospho-IRF-3 (Ser386)	Cell Signaling Technology	Cell Signaling Technology Cat# 37829, RRID:AB_2799121
Phospho-Histone H2A.X (Ser139)	Cell Signaling Technology	Cell Signaling Technology Cat# 2577, RRID:AB_2118010
CDK1 (cdc2)	Cell Signaling Technology	Cell Signaling Technology Cat# 77055, RRID:AB_2716331
Phospho-CDK1 (cdc2) (Tyr15)	Cell Signaling Technology	Cell Signaling Technology Cat# 4539, RRID:AB_560953
cGAS	Cell Signaling Technology	Cell Signaling Technology Cat# 15102, RRID:AB_2732795
Histone H3	Cell Signaling Technology	Cell Signaling Technology Cat# 4499, RRID:AB_10544537
Lamin B1	Cell Signaling Technology	Cell Signaling Technology Cat# 12586, RRID:AB_2650517
Cleaved PARP (Asp214)	Cell Signaling Technology	Cell Signaling Technology Cat# 5625, RRID:AB_10699459
Cleaved PARP (Asp214)	Cell Signaling Technology	Cell Signaling Technology Cat# 94885, RRID:AB_2800237
PD-L1	Cell Signaling Technology	Cell Signaling Technology Cat# 13684, RRID:AB_2687655
β-Actin	Cell Signaling Technology	Cell Signaling Technology Cat# 4967, RRID:AB_330288
Anti-rabbit IgG, HRP-linked Antibody	Cell Signaling Technology	Cell Signaling Technology Cat# 7074, RRID:AB_2099233
CD4-APC Cy7	Biolegend	BioLegend Cat# 100526, RRID:AB_312727
CD8-PE Cy7	Biolegend	BioLegend Cat# 100722, RRID:AB_312761
CD45-Pacific Blue	Biolegend	BioLegend Cat# 103126, RRID:AB_493535
CD4-PE/Dazzle-594	Biolegend	BioLegend Cat# 100246, RRID:AB_2565883
Ghost Dye™ Violet 510	Tonbo	Cat#13-0870-T500
PD-1-BV605	Biolegend	BioLegend Cat# 135220, RRID:AB_2562616
CD62L-AF700	BD Biosciences	BD Biosciences Cat# 560517, RRID:AB_1645210
CD44-BV711	Biolegend	BioLegend Cat# 103057, RRID:AB_2564214
Tim-3-APC	Biolegend	BioLegend Cat# 134008, RRID:AB_2562998
GR-1-BV711	Biolegend	BioLegend Cat# 108443, RRID:AB_2562549
F/80-APC	Tonbo	Tonbo Biosciences Cat# 20-4801, RRID:AB_2621602
CD11b-BV650	Biolegend	BioLegend Cat# 101259, RRID:AB_2566568
CD68-PerCP/Cy5.5	Biolegend	BioLegend Cat# 137010, RRID:AB_2260046
iNOS-PE	Thermo Fisher Scientific	Thermo Fisher Scientific Cat# 12-5920-80, RRID:AB_2572641
PD-L1-PE	BD Biosciences	BD Biosciences Cat# 558091, RRID:AB_397018
PD-L1-APC	Biolegend	BioLegend Cat# 329708, RRID:AB_940360
H-2Kb/H-2Dd-FITC	Biolegend	BioLegend Cat# 114706, RRID:AB_313605
HLA-ABC-PE	Biolegend	BioLegend Cat# 311406, RRID:AB_314875
CD3	Abcam	Abcam Cat# ab135372, RRID:AB_2884903
CD8a	Synaptic Systems	Synaptic Systems Cat# HS-361 003
PD-L1	R and D Systems	R and D Systems Cat# AF1019, RRID:AB_354540

Chemicals, peptides, and recombinant proteins		

AZD1775	Selleck chemical	Cat# S1525
H151	Selleck chemical	Cat# S6652
C176	Selleck chemical	Cat# S6575
Anti-PD-L1 antibodiy (clone 10F.9G2)	Bio X cell	Bio X Cell Cat# BE0101, RRID:AB_10949073
IgG2b I sotype control	Bio X cell	Bio X Cell Cat# BE0090, RRID:AB_1107780
Cisplatin	Accord Healthcare	Cat# 16729-288-38
Etoposide	Accord Healthcare	Cat# 16729-114-08
Recombinant Human IFN-γ	BioLegend	Cat# 570204

Critical commercial assays		

CellTiter-Glo luminescent cell viability assay	Promega	Cat# G7571
Annexin V-propidium iodide (PI) assay	BD Biosciences	BD Biosciences Cat# 556547, RRID:AB_2869082
Click-iT™ EdU Alexa Fluor™ 488 Flow Cytometry Assay Kit	Thermo Fisher	Cat# C10425
ProLong Gold Antifade Mountant with DAPI	Cell Signaling Technology	Cat# 8961S

Deposited data		

Raw RNAseq data	This paper	ArrayExpress: E-MTAB-11577

Experimental models: Cell lines		

NCI-H1048	ATCC	ATCC Cat# CRL-5853, RRID:CVCL_1453
NCI-H196	ATCC	ATCC Cat# CRL-5823, RRID:CVCL_1509
NCI-H211	ATCC	ATCC Cat# CRL-5824, RRID:CVCL_1529
NCI-H82	ATCC	ATCC Cat# HTB-175, RRID:CVCL_1591
NCI-H446	ATCC	ATCC Cat# HTB-171, RRID:CVCL_1562
NCI-H1341	ATCC	ATCC Cat# CRL-5864, RRID:CVCL_1463
NCI-H720	ATCC	ATCC Cat# CRL-5838, RRID:CVCL_1583
NCI-H524	ATCC	ATCC Cat# CRL-5831, RRID:CVCL_1568
NCI-H841	ATCC	ATCC Cat# CRL-5845, RRID:CVCL_1595
NCI-H526	ATCC	ATCC Cat# CRL-5811, RRID:CVCL_1569
NCI-H1694	ATCC	ATCC Cat# CRL-5888, RRID:CVCL_1489
NCI-H146	ATCC	ATCC Cat# HTB-173, RRID:CVCL_1473
DMS 114	ATCC	ATCC Cat# CRL-2066, RRID:CVCL_1174
COR-L311	ECACC	ECACC Cat# 96020721, RRID:CVCL_2412
NCI-H69	ATCC	ATCC Cat# HTB-119, RRID:CVCL_1579
DMS 79	ATCC	ATCC Cat# CRL-2049, RRID:CVCL_1178

Experimental models: Organisms/strains		

Athymic *nu/nu*, females, 6 weeks old	ENVIGO	Stock #: 069
B6 129F1, females, 6 weeks old	TACONIC FARMS INC.	Stock #: B6129-F
B6 FVBF1/J, females, 6 weeks old	JACKSON LABORATORY	Stock #: 019019
NSG, females, 6 weeks old	JACKSON LABORATORY	Stock #: 005557

Software and algorithms		

FlowJo Ver.10	BD Biosciences	https://www.flowjo.com
Prism Ver. 9.0	Graphpad	N/A
Case Viewer	3DHISTECH	https://www.3dhistech.com/solutions/caseviewer/

## Highlights

- WEE1 inhibition causes activation of STING and STAT1 pathways in SCLC
- WEE1 inhibitor plus ICB mediates the innate and adaptive anti-immune response
- Combined WEE1 inhibitor plus ICB suppresses tumor growth in SCLC mouse models

## References

[R1] Alexandrov LB, Nik-Zainal S, Wedge DC, Aparicio SA, Behjati S, Biankin AV, Bignell GR, Bolli N, Borg A, Børresen-Dale AL, (2013). Signatures of mutational processes in human cancer. Nature 500, 415–421. 10.1038/nature12477. 23945592 PMC3776390

[R2] Antonia SJ, López-Martin JA, Bendell J, Ott PA, Taylor M, Eder JP, Jäger D, Pietanza MC, Le DT, de Braud F, (2016). Nivolumab alone and nivolumab plus ipilimumab in recurrent small-cell lung cancer (CheckMate 032): a multicentre, open-label, phase 1/2 trial. Lancet Oncol. 17, 883–895. 10.1016/s1470-2045(16)30098-5. 27269741

[R3] Byers LA, and Rudin CM (2015). Small cell lung cancer: where do we go from here? Cancer 121, 664–672. 10.1002/cncr.29098. 25336398 PMC5497465

[R4] Byers LA, Wang J, Nilsson MB, Fujimoto J, Saintigny P, Yordy J, Giri U, Peyton M, Fan YH, Diao L, (2012). Proteomic profiling identifies dysregulated pathways in small cell lung cancer and novel therapeutic targets including PARP1. Cancer Discov. 2, 798–811. 10.1158/2159-8290.Cd-12-0112. 22961666 PMC3567922

[R5] Cargill KR, Stewart CA, Park EM, Ramkumar K, Gay CM, Cardnell RJ, Wang Q, Diao L, Shen L, Fan YH, (2021). Targeting MYC-enhanced glycolysis for the treatment of small cell lung cancer. Cancer Metab. 9, 33. 10.1186/s40170-021-00270-9. 34556188 PMC8461854

[R6] Chalishazar MD, Wait SJ, Huang F, Ireland AS, Mukhopadhyay A, Lee Y, Schuman SS, Guthrie MR, Berrett KC, Vahrenkamp JM, (2019). MYC-driven small-cell lung cancer is metabolically distinct and vulnerable to arginine depletion. Clin. Cancer Res. 25, 5107–5121. 10.1158/1078-0432.Ccr-18-4140. 31164374 PMC6697617

[R7] Dammert MA, Brägelmann J, Olsen RR, Böhm S, Monhasery N, Whitney CP, Chalishazar MD, Tumbrink HL, Guthrie MR, Klein S, (2019). MYC paralog-dependent apoptotic priming orchestrates a spectrum of vulnerabilities in small cell lung cancer. Nat. Commun. 10, 3485. 10.1038/s41467-019-11371-x. 31375684 PMC6677768

[R8] Do K, Doroshow JH, and Kummar S (2013). Wee1 kinase as a target for cancer therapy. Cell Cycle 12, 3159–3164. 10.4161/cc.26062. 24013427 PMC3865011

[R9] Do K, Wilsker D, Ji J, Zlott J, Freshwater T, Kinders RJ, Collins J, Chen AP, Doroshow JH, and Kummar S (2015). Phase I study of single- agent AZD1775 (MK-1775), a Wee1 kinase inhibitor, in patients with refractory solid tumors. J. Clin. Oncol. 33, 3409–3415. 10.1200/jco.2014.60.4009. 25964244 PMC4606059

[R10] Eerola AK, Soini Y, and Pääkkö P (2000). A high number of tumor-infiltrating lymphocytes are associated with a small tumor size, low tumor stage, and a favorable prognosis in operated small cell lung carcinoma. Clin. Cancer Res. 6, 1875–1881. 10815910

[R11] Färkkilä A, Gulhan DC, Casado J, Jacobson CA, Nguyen H, Kochupurakkal B, Maliga Z, Yapp C, Chen YA, Schapiro D, (2020). Immunogenomic profiling determines responses to combined PARP and PD-1 inhibition in ovarian cancer. Nat. Commun. 11, 1459. 10.1038/s41467-020-15315-8. 32193378 PMC7081234

[R12] Foy V, Schenk MW, Baker K, Gomes F, Lallo A, Frese KK, Forster M, Dive C, and Blackhall F (2017). Targeting DNA damage in SCLC. Lung Cancer 114, 12–22. 10.1016/j.lungcan.2017.10.006. 29173760

[R13] Fumet JD, Limagne E, Thibaudin M, Truntzer C, Bertaut A, Rederstorff E, and Ghiringhelli F (2020). Precision medicine phase II study evaluating the efficacy of a double immunotherapy by durvalumab and tremelimumab combined with olaparib in patients with solid cancers and carriers of homologous recombination repair genes mutation in response or stable after olaparib treatment. BMC Cancer 20, 748. 10.1186/s12885-020-07253-x. 32778095 PMC7418426

[R14] Garcia-Diaz A, Shin DS, Moreno BH, Saco J, Escuin-Ordinas H, Rodriguez GA, Zaretsky JM, Sun L, Hugo W, Wang X, (2017). Interferon receptor signaling pathways regulating PD-L1 and PD-L2 expression. Cell Rep. 19, 1189–1201. 10.1016/j.celrep.2017.04.031. 28494868 PMC6420824

[R15] George J, Lim JS, Jang SJ, Cun Y, Ozretić L, Kong G, Leenders F, Lu X, Fernández-Cuesta L, Bosco G, (2015). Comprehensive genomic profiles of small cell lung cancer. Nature 524, 47–53. 10.1038/nature14664. 26168399 PMC4861069

[R16] Gocher AM, Workman CJ, and Vignali DAA (2021). Interferon-γ: teammate or opponent in the tumour microenvironment? Nat. Rev. Immunol. 22, 158–172. 10.1038/s41577-021-00566-3. 34155388 PMC8688586

[R17] Higuchi T, Flies DB, Marjon NA, Mantia-Smaldone G, Ronner L, Gimotty PA, and Adams SF (2015). CTLA-4 blockade synergizes therapeutically with PARP inhibition in BRCA1-deficient ovarian cancer. Cancer Immunol. Res. 3, 1257–1268. 10.1158/2326-6066.Cir-15-0044. 26138335 PMC4984269

[R18] Horn L, Mansfield AS, Szczę sna A, Havel L, Krzakowski M, Hochmair MJ, Huemer F, Losonczy G, Johnson ML, Nishio M, (2018). First-line atezolizumab plus chemotherapy in extensive-stage small-cell lung cancer. N. Engl. J. Med. 379, 2220–2229. 10.1056/NEJMoa1809064. 30280641

[R19] Huang J, Wang L, Cong Z, Amoozgar Z, Kiner E, Xing D, Orsulic S, Matulonis U, and Goldberg MS (2015). The PARP1 inhibitor BMN 673 exhibits immunoregulatory effects in a Brca1(−/−) murine model of ovarian cancer. Biochem. Biophys. Res. Commun. 463, 551–556. 10.1016/j.bbrc.2015.05.083. 26047697

[R20] Ireland AS, Micinski AM, Kastner DW, Guo B, Wait SJ, Spainhower KB, Conley CC, Chen OS, Guthrie MR, Soltero D, (2020). MYC drives temporal evolution of small cell lung cancer subtypes by reprogramming neuroendocrine fate. Cancer Cell 38, 60–78.e12. 10.1016/j.ccell.2020.05.001. 32473656 PMC7393942

[R21] Le DT, Uram JN, Wang H, Bartlett BR, Kemberling H, Eyring AD, Skora AD, Luber BS, Crocenzi TS, Fisher GA, (2015). PD-1 blockade in tumors with mismatch-repair deficiency. N. Engl. J. Med. 372, 2509–2520. 10.1056/NEJMoa1500596. 26028255 PMC4481136

[R22] Leijen S, van Geel RM, Pavlick AC, Tibes R, Rosen L, Razak ARA, Lam R, Demuth T, Rose S, Lee MA, (2016a). Phase I study evaluating WEE1 inhibitor AZD1775 as monotherapy and in combination with gemcitabine, cisplatin, or carboplatin in patients with advanced solid tumors. J. Clin. Oncol. 34, 4371–4380. 10.1200/jco.2016.67.5991. 27601554 PMC7845944

[R23] Leijen S, van Geel RM, Sonke GS, de Jong D, Rosenberg EH, Marchetti S, Pluim D, van Werkhoven E, Rose S, Lee MA, (2016b). Phase II study of WEE1 inhibitor AZD1775 plus carboplatin in patients with TP53-mutated ovarian cancer refractory or resistant to first-line therapy within 3 months. J. Clin. Oncol. 34, 4354–4361. 10.1200/jco.2016.67.5942. 27998224

[R24] Liberzon A, Subramanian A, Pinchback R, Thorvaldsdóttir H, Tamayo P, and Mesirov JP (2011). Molecular signatures database (MSigDB) 3.0. Bioinformatics 27, 1739–1740. 10.1093/bioinformatics/btr260. 21546393 PMC3106198

[R25] Liu SV, Reck M, Mansfield AS, Mok T, Scherpereel A, Reinmuth N, Garassino MC, De Castro Carpeno J, Califano R, Nishio M, (2021). Updated overall survival and PD-L1 subgroup Analysis of patients with extensive-stage small-cell lung cancer treated with atezolizumab, carboplatin, and etoposide (IMpower133). J. Clin. Oncol. 39, 619–630. 10.1200/jco.20.01055. 33439693 PMC8078320

[R26] Michalska A, Blaszczyk K, Wesoly J, and Bluyssen HAR (2018). A positive feedback amplifier circuit that regulates interferon (IFN)-Stimulated gene expression and controls type I and type II IFN responses. Front. Immunol. 9, 1135. 10.3389/fimmu.2018.01135. 29892288 PMC5985295

[R27] Mollaoglu G, Guthrie MR, Böhm S, Brägelmann J, Can I, Ballieu PM, Marx A, George J, Heinen C, Chalishazar MD, (2017). MYC drives progression of small cell lung cancer to a variant neuroendocrine subtype with vulnerability to Aurora kinase inhibition. Cancer Cell 31, 270–285. 10.1016/j.ccell.2016.12.005. 28089889 PMC5310991

[R28] Morel KL, Sheahan AV, Burkhart DL, Baca SC, Boufaied N, Liu Y, Qiu X, Cañadas I, Roehle K, Heckler M, (2021). EZH2 inhibition activates a dsRNA-STING-interferon stress axis that potentiates response to PD-1 checkpoint blockade in prostate cancer. Nat. Cancer 2, 444–456. 10.1038/s43018-021-00185-w. 33899001 PMC8061902

[R29] Ott PA, Elez E, Hiret S, Kim DW, Morosky A, Saraf S, Piperdi B, and Mehnert JM (2017). Pembrolizumab in patients with extensive-stage small-cell lung cancer: results from the phase Ib KEYNOTE-028 study. J. Clin. Oncol. 35, 3823–3829. 10.1200/jco.2017.72.5069. 28813164

[R30] Oza AM, Estevez-Diz M, Grischke EM, Hall M, Marmé F, Provencher D, Uyar D, Weberpals JI, Wenham RM, Laing N, (2020). A biomarker-enriched, randomized phase II trial of adavosertib (AZD1775) plus paclitaxel and carboplatin for women with platinum-sensitive TP53-mutant ovarian cancer. Clin. Cancer Res. 26, 4767–4776. 10.1158/1078-0432.Ccr-20-0219. 32611648

[R31] Patro R, Duggal G, Love MI, Irizarry RA, and Kingsford C (2017). Salmon provides fast and bias-aware quantification of transcript expression. Nat. Methods 14, 417–419. 10.1038/nmeth.4197. 28263959 PMC5600148

[R32] Paz-Ares L, Dvorkin M, Chen Y, Reinmuth N, Hotta K, Trukhin D, Statsenko G, Hochmair MJ, Özgüroğlu M, Ji JH, (2019). Durvalumab plus platinum-etoposide versus platinum-etoposide in first-line treatment of extensive-stage small-cell lung cancer (CASPIAN): a randomised, controlled, open-label, phase 3 trial. Lancet 394, 1929–1939. 10.1016/s0140-6736(19)32222-6. 31590988

[R33] Pimentel H, Bray NL, Puente S, Melsted P, and Pachter L (2017). Differential analysis of RNA-seq incorporating quantification uncertainty. Nat. Methods 14, 687–690. 10.1038/nmeth.4324. 28581496

[R34] Poirier JT, George J, Owonikoko TK, Berns A, Brambilla E, Byers LA, Carbone D, Chen HJ, Christensen CL, Dive C, (2020). New approaches to SCLC therapy: from the laboratory to the clinic. J. Thorac. Oncol. 15, 520–540. 10.1016/j.jtho.2020.01.016. 32018053 PMC7263769

[R35] PosthumaDeBoer J, Würdinger T, Graat HC, van Beusechem VW, Helder MN, van Royen BJ, and Kaspers GJ (2011). WEE1 inhibition sensitizes osteosarcoma to radiotherapy. BMC Cancer 11, 156. 10.1186/1471-2407-11-156. 21529352 PMC3103478

[R36] Rudin CM, Durinck S, Stawiski EW, Poirier JT, Modrusan Z, Shames DS, Bergbower EA, Guan Y, Shin J, Guillory J, (2012). Comprehensive genomic analysis identifies SOX2 as a frequently amplified gene in small-cell lung cancer. Nat. Genet. 44, 1111–1116. 10.1038/ng.2405. 22941189 PMC3557461

[R37] Rudin CM, Poirier JT, Byers LA, Dive C, Dowlati A, George J, Heymach JV, Johnson JE, Lehman JM, MacPherson D, (2019). Molecular subtypes of small cell lung cancer: a synthesis of human and mouse model data. Nat. Rev. Cancer 19, 289–297. 10.1038/s41568-019-0133-9. 30926931 PMC6538259

[R38] Schaffer BE, Park KS, Yiu G, Conklin JF, Lin C, Burkhart DL, Karnezis AN, Sweet-Cordero EA, and Sage J (2010). Loss of p130 accelerates tumor development in a mouse model for human small-cell lung carcinoma. Cancer Res. 70, 3877–3883. 10.1158/0008-5472.Can-09-4228. 20406986 PMC2873158

[R39] Seligmann JF, Fisher DJ, Brown LC, Adams RA, Graham J, Quirke P, Richman SD, Butler R, Domingo E, Blake A, (2021). Inhibition of WEE1 is effective in TP53- and RAS-mutant metastatic colorectal cancer: a randomized trial (FOCUS4-C) comparing adavosertib (AZD1775) with active monitoring. J. Clin. Oncol. 39, 3705–3715, Jco2101435. 10.1200/jco.21.01435. 34538072 PMC8601321

[R40] Sen T, Tong P, Diao L, Li L, Fan Y, Hoff J, Heymach JV, Wang J, and Byers LA (2017a). Targeting AXL and mTOR pathway overcomes primary and acquired resistance to WEE1 inhibition in small-cell lung cancer. Clin. Can- cer Res. 23, 6239–6253. 10.1158/1078-0432.Ccr-17-1284. PMC5882197 28698200

[R41] Sen T, Tong P, Stewart CA, Cristea S, Valliani A, Shames DS, Redwood AB, Fan YH, Li L, Glisson BS, (2017b). CHK1 inhibition in small-cell lung cancer produces single-agent activity in biomarker-defined dis- ease subsets and combination activity with cisplatin or olaparib. Cancer Res. 77, 3870–3884. 10.1158/0008-5472.Can-16-3409. 28490518 PMC5563854

[R42] Sen T, Gay CM, and Byers LA (2018). Targeting DNA damage repair in small cell lung cancer and the biomarker landscape. Transl. Lung Cancer Res. 7, 50–68. 10.21037/tlcr.2018.02.03. 29535912 PMC5835589

[R43] Sen T, Della Corte CM, Milutinovic S, Cardnell RJ, Diao L, Ramkumar K, Gay CM, Stewart CA, Fan Y, Shen L, (2019a). Combination treatment of the oral CHK1 inhibitor, SRA737, and low-dose gemcitabine enhances the effect of programmed death ligand 1 blockade by modulating the immune microenvironment in SCLC. J. Thorac. Oncol. 14, 2152–2163. 10.1016/j.jtho.2019.08.009. 31470128 PMC7141083

[R44] Sen T, Rodriguez BL, Chen L, Corte CMD, Morikawa N, Fujimoto J, Cristea S, Nguyen T, Diao L, Li L, (2019b). Targeting DNA damage response promotes antitumor immunity through STING-mediated T-cell activation in small cell lung cancer. Cancer Discov. 9, 646–661. 10.1158/2159-8290.Cd-18-1020. 30777870 PMC6563834

[R45] Sos ML, Dietlein F, Peifer M, Schöttle J, Balke-Want H, Müller C, Koker M, Richters A, Heynck S, Malchers F, (2012). A framework for identification of actionable cancer genome dependencies in small cell lung cancer. Proc. Natl. Acad. Sci. U S A 109, 17034–17039. 10.1073/pnas.1207310109. 23035247 PMC3479457

[R46] Subramanian A, Tamayo P, Mootha VK, Mukherjee S, Ebert BL, Gillette MA, Paulovich A, Pomeroy SL, Golub TR, Lander ES, and Mesirov JP (2005). Gene set enrichment analysis: a knowledge-based approach for interpreting genome-wide expression profiles. Proc. Natl. Acad. Sci. U S A 102, 15545–15550. 10.1073/pnas.0506580102. 16199517 PMC1239896

[R47] Sun W, Zhang Q, Wang R, Li Y, Sun Y, and Yang L (2021). Targeting DNA damage repair for immune checkpoint inhibition: mechanisms and potential clinical applications. Front. Oncol. 11, 648687. 10.3389/fonc.2021.648687. PMC8137908 34026622

[R48] Takahashi M, Lio CWJ, Campeau A, Steger M, Ay F, Mann M, Gonzalez DJ, Jain M, and Sharma S (2021). The tumor suppressor kinase DAPK3 drives tumor-intrinsic immunity through the STING-IFN-β pathway. Nat. Immunol. 22, 485–496. 10.1038/s41590-021-00896-3. 33767426 PMC8300883

[R49] Taniguchi H, Sen T, and Rudin CM (2020). Targeted therapies and biomarkers in small cell lung cancer. Front. Oncol. 10, 741. 10.3389/fonc.2020.00741. 32509576 PMC7251180

[R50] Teo MY, Seier K, Ostrovnaya I, Regazzi AM, Kania BE, Moran MM, Cipolla CK, Bluth MJ, Chaim J, Al-Ahmadie H, (2018). Alterations in DNA damage response and repair genes as potential marker of clinical benefit from PD-1/PD-L1 blockade in advanced urothelial cancers. J. Clin. Oncol. 36, 1685–1694. 10.1200/jco.2017.75.7740. 29489427 PMC6366295

[R51] Wang Y, Li J, Booher RN, Kraker A, Lawrence T, Leopold WR, and Sun Y (2001). Radiosensitization of p53 mutant cells by PD0166285, a novel G(2) checkpoint abrogator. Cancer Res. 61, 8211–8217. 11719452

[R52] Wang Y, Decker SJ, and Sebolt-Leopold J (2004). Knockdown of Chk1, Wee1 and Myt1 by RNA interference abrogates G2 checkpoint and induces apoptosis. Cancer Biol. Ther. 3, 305–313. 10.4161/cbt.3.3.697. 14726685

[R53] Wohlhieter CA, Richards AL, Uddin F, Hulton CH, Quintanal-Villalonga À, Martin A, de Stanchina E, Bhanot U, Asher M, Shah NS, (2020). Concurrent mutations in STK11 and KEAP1 promote ferroptosis protection and SCD1 dependence in lung cancer. Cell Rep. 33, 108444. 10.1016/j.celrep.2020.108444. PMC7722473 33264619

[R54] Woo SR, Corrales L, and Gajewski TF (2015). The STING pathway and the T cell-inflamed tumor microenvironment. Trends Immunol. 36, 250–256. 10.1016/j.it.2015.02.003. 25758021 PMC4393801

[R55] Wu X, Kang X, Zhang X, Xie W, Su Y, Liu X, Guo L, Guo E, Li F, Hu D, (2021a). WEE1 inhibitor and ataxia telangiectasia and RAD3-related inhibitor trigger stimulator of interferon gene-dependent immune response and enhance tumor treatment efficacy through programmed death-ligand 1 blockade. Cancer Sci. 112, 4444–4456. 10.1111/cas.15108. 34382294 PMC8586668

[R56] Wu X, Nelson M, Basu M, Srinivasan P, Lazarski C, Zhang P, Zheng P, and Sandler AD (2021b). MYC oncogene is associated with suppression of tumor immunity and targeting Myc induces tumor cell immunogenicity for therapeutic whole cell vaccination. J. Immunother. Cancer 9, e001388. 10.1136/jitc-2020-001388. PMC7993333 33757986

[R57] Yu G, Wang LG, Han Y, and He QY (2012). clusterProfiler: an R package for comparing biological themes among gene clusters. OMICS 16, 284–287. 10.1089/omi.2011.0118. 22455463 PMC3339379

[R58] Zerbino DR, Achuthan P, Akanni W, Amode MR, Barrell D, Bhai J, Billis K, Cummins C, Gall A, Girón CG, (2018). Ensembl 2018. Nucleic Acids Res. 46, D754–d761. 10.1093/nar/gkx1098. 29155950 PMC5753206

[R59] Zhang H, Christensen CL, Dries R, Oser MG, Deng J, Diskin B, Li F, Pan Y, Zhang X, Yin Y, (2020). CDK7 inhibition potentiates genome Instability triggering anti-tumor immunity in small cell lung cancer. Cancer Cell 37, 37–54.e9. 10.1016/j.ccell.2019.11.003. 31883968 PMC7277075

[R60] Zheng J, Mo J, Zhu T, Zhuo W, Yi Y, Hu S, Yin J, Zhang W, Zhou H, and Liu Z (2020). Comprehensive elaboration of the cGAS-STING signaling axis in cancer development and immunotherapy. Mol. Cancer 19, 133. 10.1186/s12943-020-01250-1. 32854711 PMC7450153

