# Supplementary material for: WEE1 inhibition enhances the antitumor immune response to PD-L1 blockade by the concomitant activation of STING and STAT1 pathways in SCLC
Source: Cell Rep. Author manuscript; Available in PMC 2022 Sep 7. (PMC9449677; doi:10.1016/j.celrep.2022.110814)
Supplement: 1 [file NIHMS1808947-supplement-1.pdf]

Cell Reports, Volume *39*

Supplemental information

WEE1 inhibition enhances the antitumor immune response to PD-L1 blockade by the concomitant activation of STING and STAT1 pathways in SCLC

Hirokazu Taniguchi, Rebecca Caeser, Shweta S. Chavan, Yingqian A. Zhan, Andrew Chow, Parvathy Manoj, Fathema Uddin, Hidenori Kitai, Rui Qu, Omar Hayatt, Nisargbhai S. Shah, Álvaro Quintanal Villalonga, Viola Allaj, Evelyn M. Nguyen, Joseph Chan, Adam O. Michel, Hiroshi Mukae, Elisa de Stanchina, Charles M. Rudin, and Triparna Sen

Figure S1:

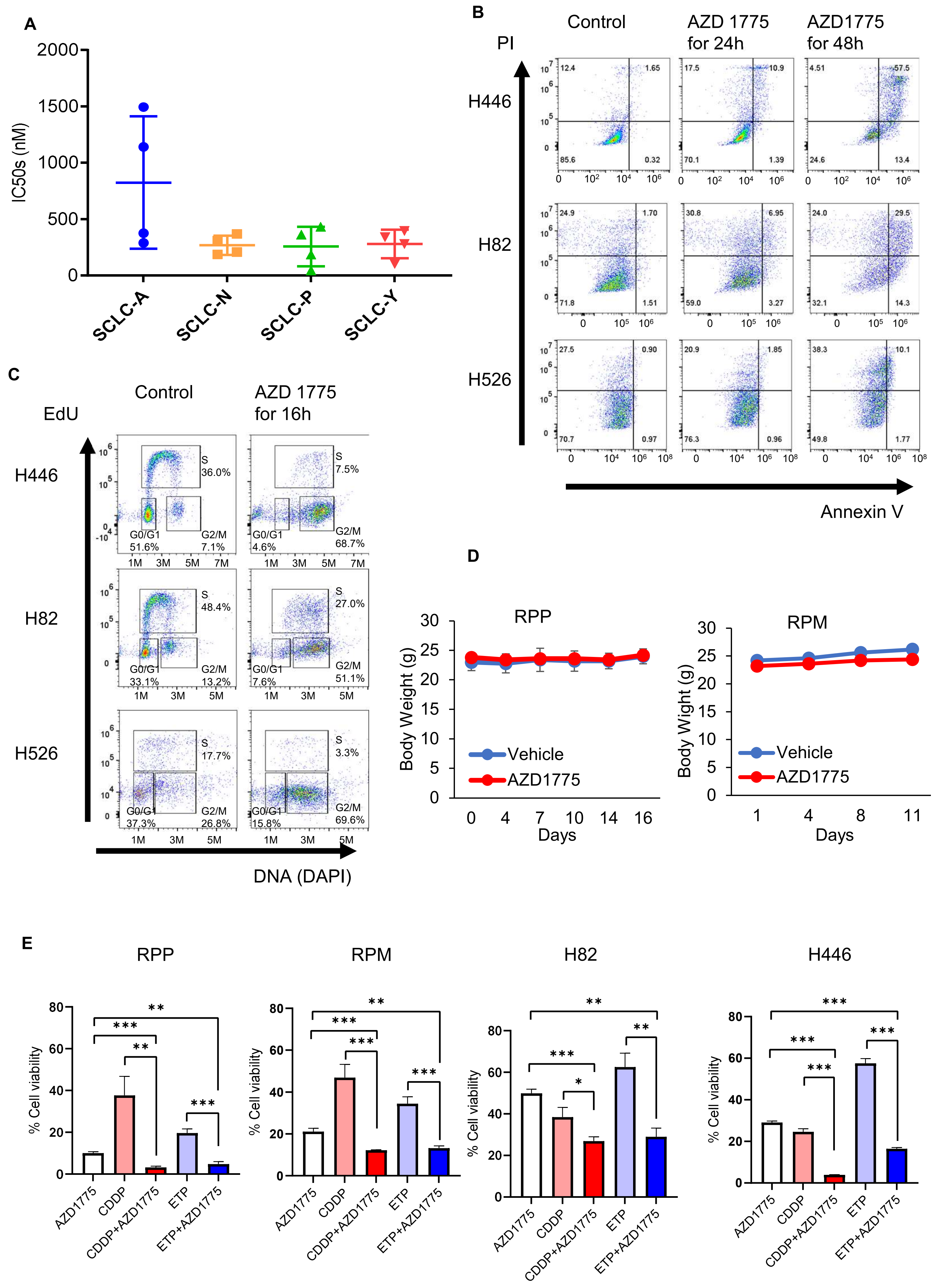
A
IC50s (nM)
2000
1500
1000
500
0
SCLC-A
SCLC-N
SCLC-P
SCLC-Y
B
PI
Control
AZD 1775 for 24h
AZD1775 for 48h
H446
H82
H526
Annexin V
C
EdU
Control
AZD 1775 for 16h
H446
H82
H526
DNA (DAPI)
D
RPP
RPM
Body Weight (g)
Body Wight (g)
Vehicle
AZD1775
Days
E
RPP
RPM
H82
H446
% Cell viability
AZD1775
CDDP
CDDP+AZD1775
ETP
ETP+AZD1775

Figure S1. The effect of AZD1775 in SCLC cells, Related to Figure 1.

(A) IC_{50} values from Fig.1A grouped by SCLC subtype. Each dot represents the mean IC_{50} value of one cell line. (B) Representative flow cytometry plots of annexin V-PI assay staining in H446, H82, H526 cells treated with 1 μM AZD1775 for 24 or 48 hours. (C) Representative plots of EdU-DAPI based flow cytometry in H82, H446, H526 cells treated with 1 μM AZD1775 for 16 hours. (D) Body weight changes of nude mice treated with vehicle or 60 mg/kg of AZD1775 (n = 5). Bars represent mean ± SD. Statistical significance was determined using Student's t-test. (E) The RPP and RPM cells were incubated with 0.5 μM AZD1775, 3 μM cisplatin (CDDP) and/or 0.3 μM etoposide (ETP) for 72 h, the H82 and H446 cells were incubated with 0.3 μM AZD1775, 3 μM cisplatin (CDDP) and/or 0.3 μM etoposide (ETP) for 72 h, The cell viability was determined using CellTiter-Glo luminescent cell viability assay. Percentages were normalized to DMSO control and bars represent mean ± SD of triplicate. Statistical significance was determined using Student's t-test ($*p < 0.05$, $**p < 0.01$, $***p < 0.001$).

Figure S2:

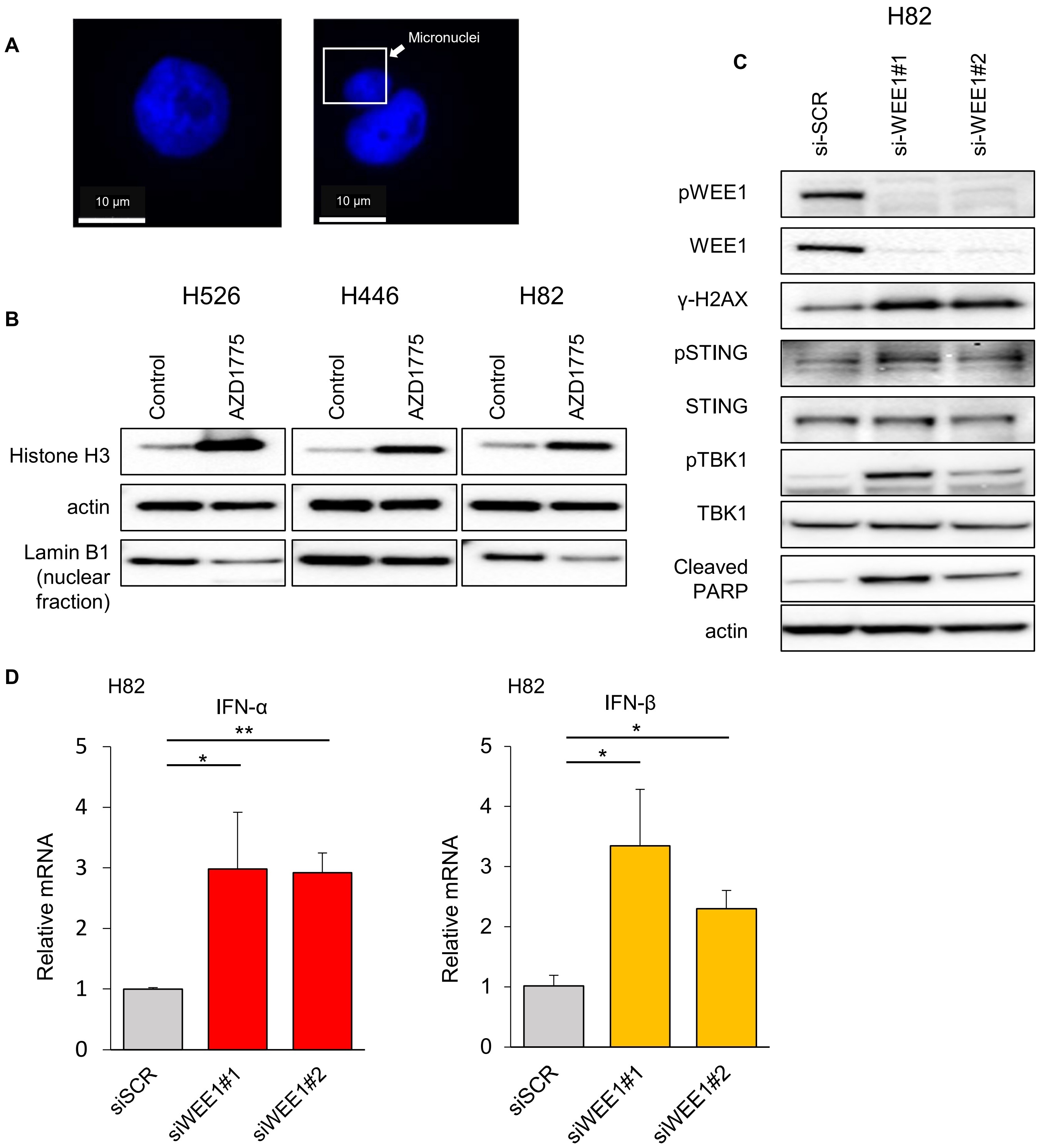
A
Micronuclei
10 µm
10 µm
B
H526
H446
H82
Control
AZD1775
Control
AZD1775
Control
AZD1775
Histone H3
actin
Lamin B1 (nuclear fraction)
C
H82
si-SCR
si-WEE1#1
si-WEE1#2
pWEE1
WEE1
γ-H2AX
pSTING
STING
pTBK1
TBK1
Cleaved PARP
actin
D
H82
IFN-α
Relative mRNA
0
1
2
3
4
5
**
*
siSCR
siWEE1#1
siWEE1#2
H82
IFN-β
Relative mRNA
*
*
siSCR
siWEE1#1
siWEE1#2

Figure S2. DNA damage induced by AZD1775 and the activation of the STING pathway by genetic knockdown of WEE1 with siRNA, Related to Figure 2.

(A) Representative images of DAPI-stained H82 cells showing micronuclei after 1 μM AZD1775 treatment for 24 hours (Right), as compared to untreated control (Left). White box and arrow indicate a typical micronuclei formation. (B) Immunoblot of histone H3 in the cytosolic fraction of SCLC cells treated with 1 μM AZD1775 for 48 hours. Nuclear Lamin B1 shows the degree of fractionation achieved. (C-D) WEE1 was knocked down using WEE1-specific siRNAs (#1 and #2) or nonspecific siRNA control (si-SCR). (C) Western blot showing expression of phospho (p)- and total (t)-WEE1, γH2AX, p- and t-STING, p- and t-TBK1, cleaved PARP, and actin (loading control) in H82 cells following knockdown. (D) Quantitative mRNA expression of IFN-α, IFN-β. The bars represent mean ± SD of triplicate. Statistical significance was determined using Student's t-test (∗p < 0.05, ∗∗p < 0.01, ∗∗∗p < 0.001).

Figure S3:

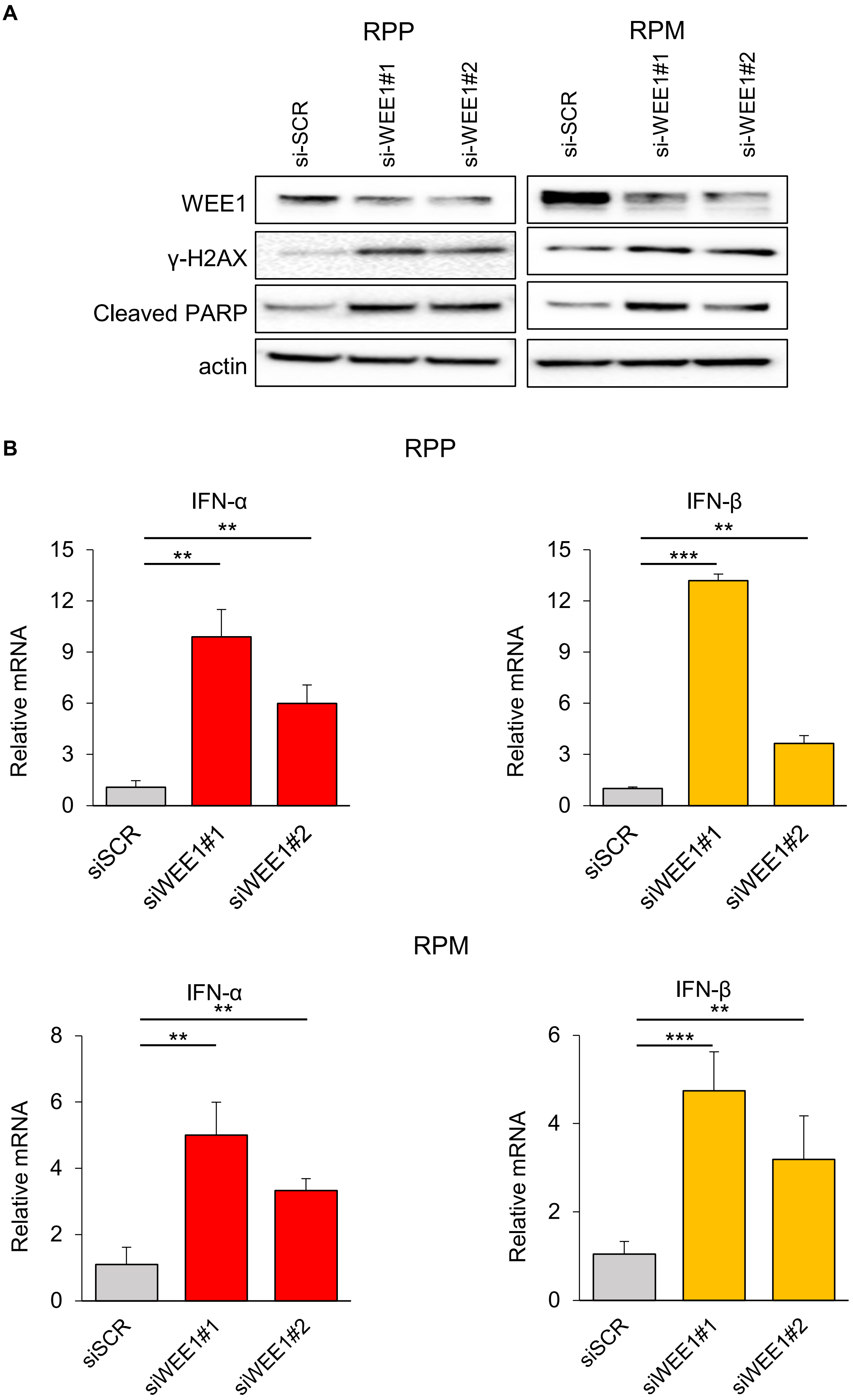

A
RPP
RPM
si-SCR
si-WEE1#1
si-WEE1#2
WEE1
γ-H2AX
Cleaved PARP
actin
B
RPP
IFN-α
IFN-β
Relative mRNA
siSCR
siWEE1#1
siWEE1#2
**
***
RPM

Figure S3. siRNA Knockdown of WEE1 induces mRNA expression of type I IFNs in RPP and RPM cells, Related to Figure 2.

Knockdown of WEE1 with WEE1-specific siRNAs (#1 and #2) or nonspecific siRNA control. (A) Western blot showing expression of total-WEE1, γH2AX, cleaved PARP, and actin (loading control) in RPP cells and RPM cells. (B) Quantitative mRNA expression of *IFN-α, IFN-β* in RPP and RPM cells following knockdown. The bars represent mean ± SD of triplicate. Statistical significance was determined using Student's t-test (*$p < 0.05$, **$p < 0.01$, ***$p < 0.001$).

Figure S4:

A

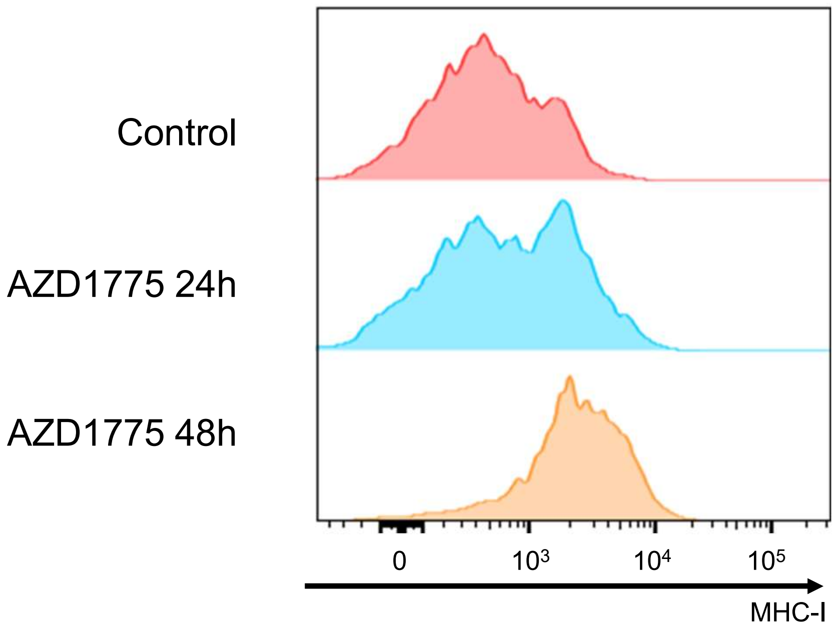
Control
AZD1775 24h
AZD1775 48h
0
10³
10⁴
10⁵
MHC-I

B

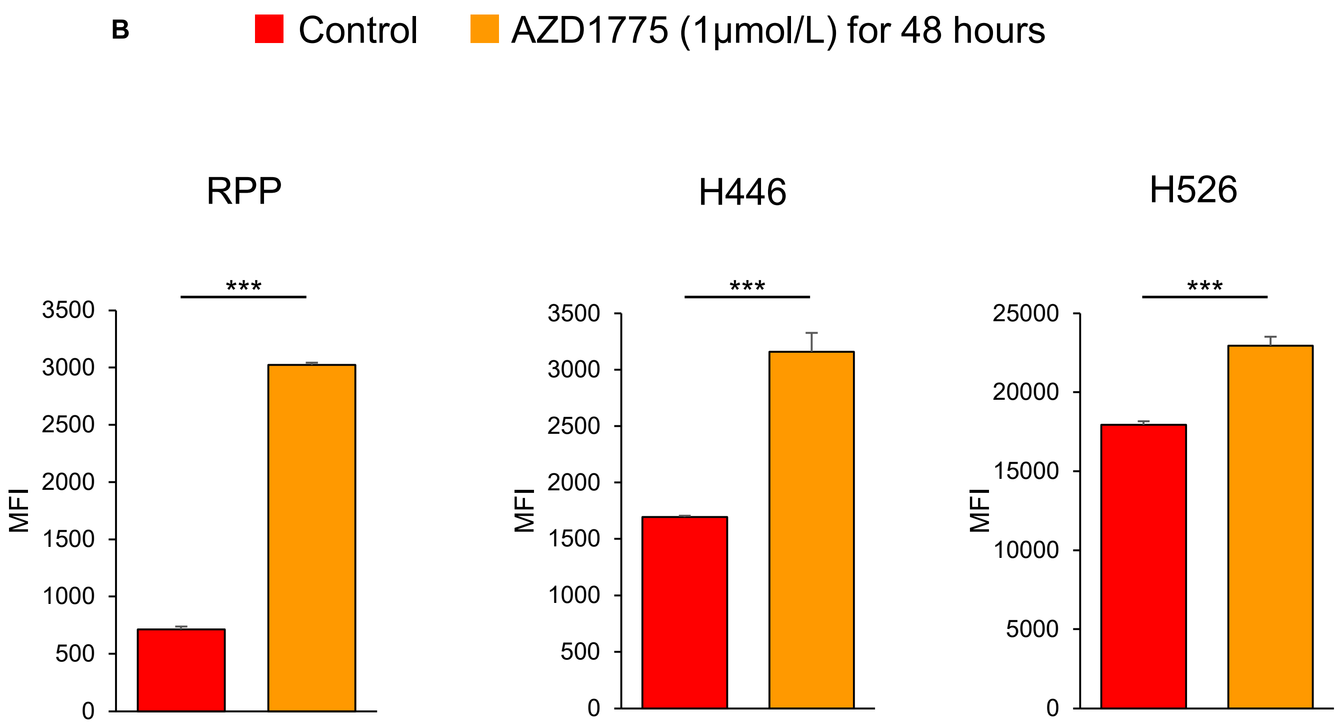
Control
AZD1775 (1μmol/L) for 48 hours
RPP
H446
H526
***
***
***
MFI
MFI
MFI
3500
3000
2500
2000
1500
1000
500
0
3500
3000
2500
2000
1500
1000
500
0
25000
20000
15000
10000
5000
0

Figure S4. WEE1 inhibitor induces the cell surface MHC-I expression, Related to Figure 2.

(A) Representative histogram of MHC-I expression in RPP cells tested by flow cytometry following 24- or 48-hour treatment with 1μM AZD1775. (B) MFI of MHC-I in SCLC (RPP, H446, H526) treated with 1μM AZD6738 for 48 hours. The bars represent mean ± SD of triplicate. Statistical significance was determined using Student's t-test (***$p < 0.001$).

Figure S5:

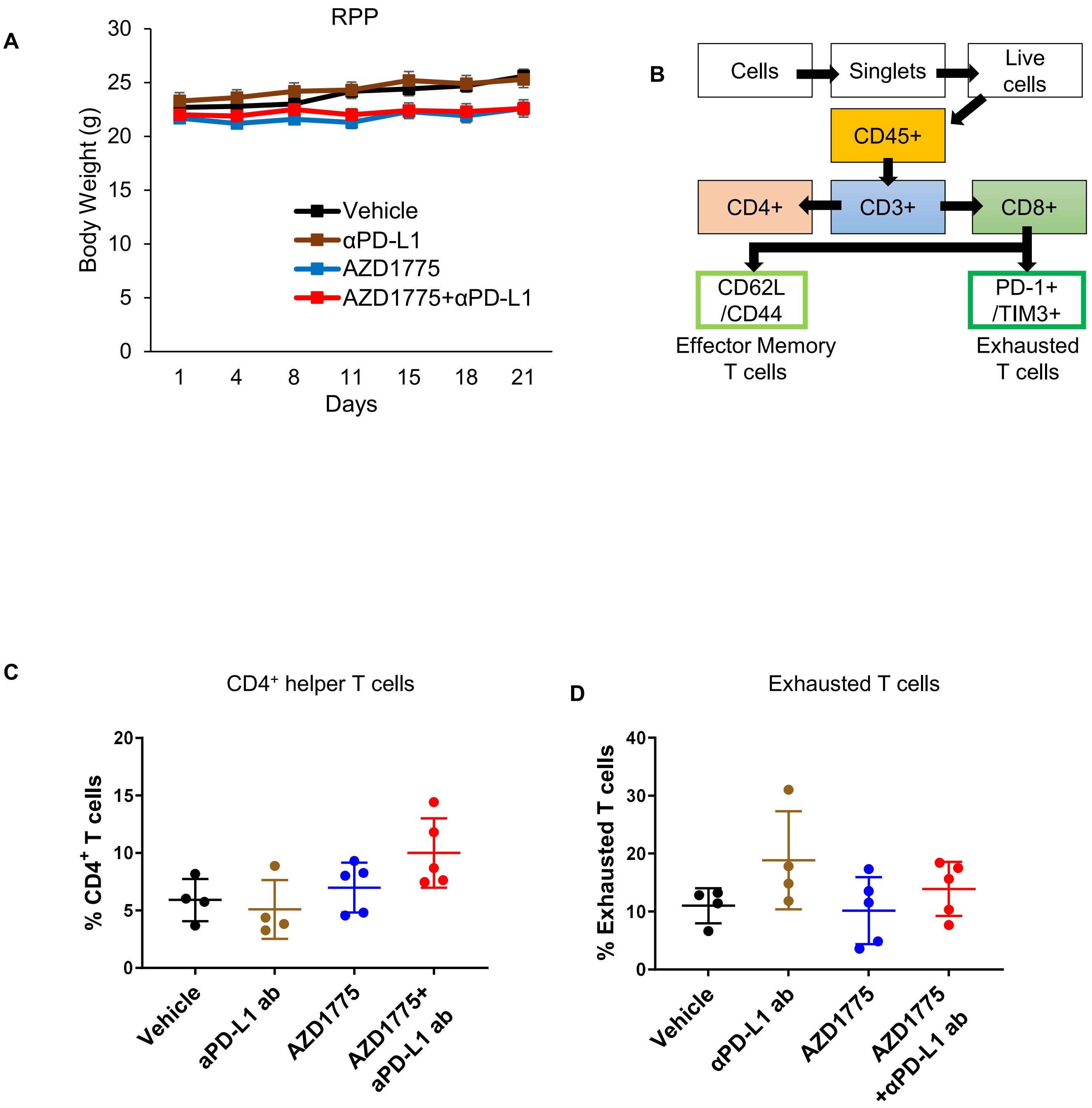
A
RPP
Body Weight (g)
30
25
20
15
10
5
0
1
4
8
11
15
18
21
Days
Vehicle
αPD-L1
AZD1775
AZD1775+αPD-L1
B
Cells
Singlets
Live cells
CD45+
CD4+
CD3+
CD8+
CD62L /CD44
Effector Memory T cells
PD-1+ /TIM3+
Exhausted T cells
C
CD4+ helper T cells
% CD4+ T cells
20
15
10
5
0
Vehicle
aPD-L1 ab
AZD1775
AZD1775+ aPD-L1 ab
D
Exhausted T cells
% Exhausted T cells
40
30
20
10
0
Vehicle
αPD-L1 ab
AZD1775
AZD1775 +αPD-L1 ab

Figure S5. WEE1 inhibition enhances antitumor responses induced by anti-PD-L1 antibody in the *in vivo* RPP model, Related to Figure 3.

(A) Body weight curves the means ± SEM of vehicle, AZD1775 alone (60 mg/kg, 5 of 7 days, Q.D.), anti-PD-L1 antibody alone (300 μg/body, 1 of 7 days), and AZD1775 plus anti-PD-L1 antibody groups in B6 129F1 mice injected with RPP cells (*n* =10 per groups). (B) Gating strategy of tumors flow cytometry analysis. (C-D) Tumors in Fig. 3A were harvested on day 15 for immune profiling by flow cytometry. Cumulative data for the tumors are shown. Flow cytometry analysis of (C) $CD45^+CD3^+CD4^+$ T cells and (D) $CD45^+CD3^+CD8^+PD\text{-}1^+TIM3^+$ T cells. Percentages in C were the ratio to $CD45^+$ cells. Percentages in D were the ratio to $CD8^+$ cells. (*n* = 5 for Vehicle and anti-PD-L1 group, *n* = 6 for AZD1775 and AZD1775+anti-PD-L1 group). The bars represent mean ± SD of triplicate. Statistical significance was determined using Student's t-test (without bars; not significant).

Figure S6:

A

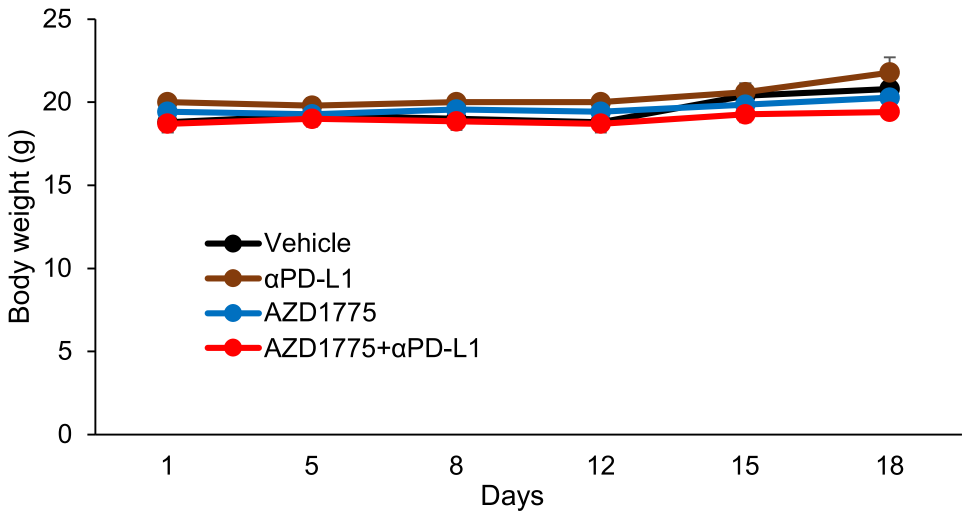

B

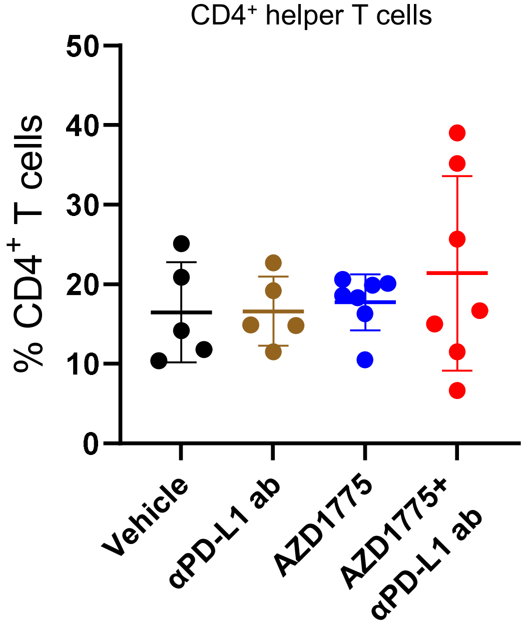

C

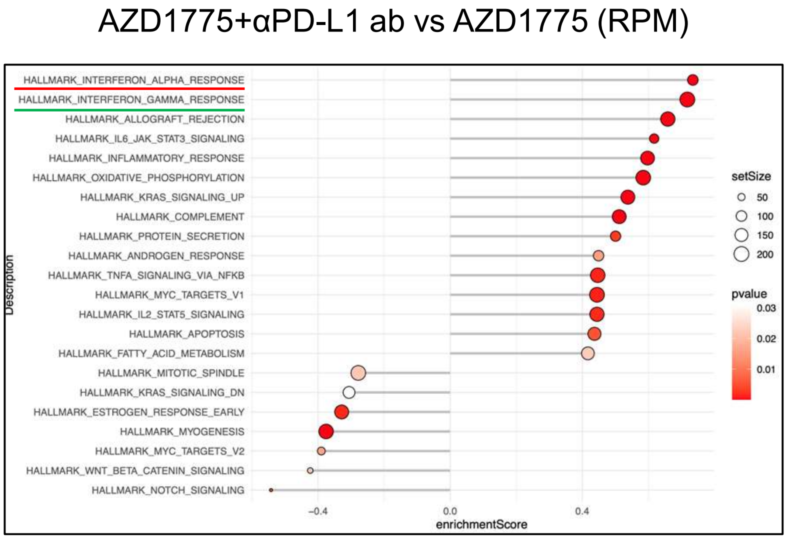

Figure S6. WEE1 inhibition enhances antitumor responses induced by anti-PD-L1 antibody in the *in vivo* RPM model, Related to Figure 4.

(A) Body weight curves the means ± SE from vehicle, AZD1775 alone (60 mg/kg, 5 of 7 days, Q.D.), anti-PD-L1 antibody alone (300 μg, 1 of 7 days), and AZD1775 plus anti-PD-L1 antibody groups in B6FVBF1/J mice injected with RPM cells (*n* = 5 for vehicle and anti-PD-L1 groups, *n* = 7 for AZD1775 and AZD1775 plus anti-PD-L1 groups). (B) Tumors in Fig. 4A were harvested on day 19 for immune profiling by flow cytometry of $CD45^{+}CD3^{+}CD4^{+}$ T cells. Cumulative data for the tumors are shown. Percentages were ratio to $CD45^{+}$ cells (*n* = 5 for Vehicle and anti-PD-L1 group, *n* = 7 for AZD1775 and AZD1775+anti-PD-L1 group). The bars represent mean ± SD of triplicate. Statistical significance was determined using Student's t-test (without bars; not significant). (C) HALLMARK pathway enrichment analyses of differentially expressed genes (DEGs) from RPM-tumors on the mice treated with AZD1775 or AZD1775 plus anti-PD-L1 antibody for 21 days. The data shown AZD1775 plus anti-PD-L1 antibody versus AZD1775 (*n* = 5 per group).

Figure S7:

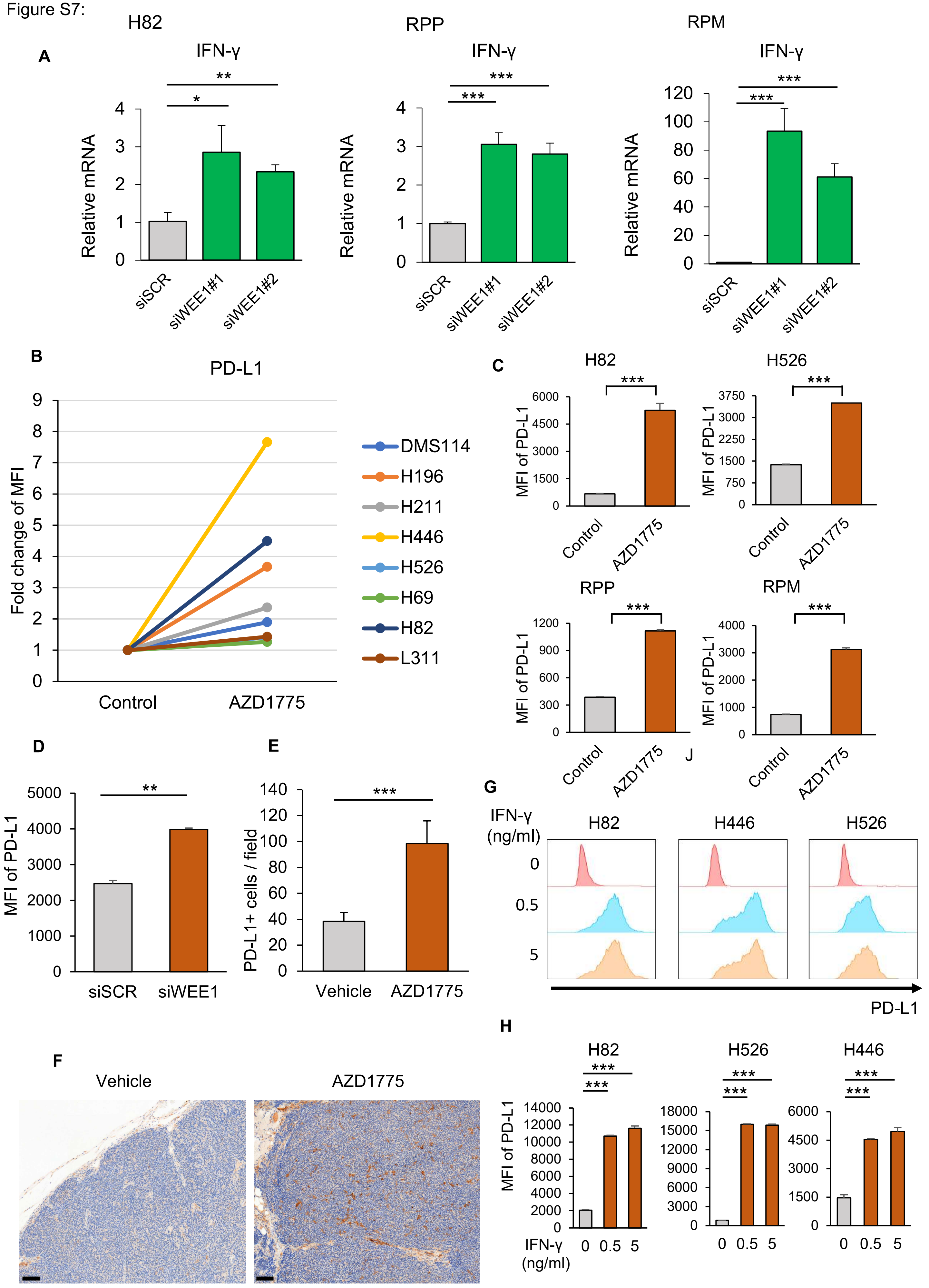
A
H82
IFN-γ
Relative mRNA
siSCR
siWEE1#1
siWEE1#2
RPP
RPM
B
PD-L1
Fold change of MFI
Control
AZD1775
DMS114
H196
H211
H446
H526
H69
H82
L311
C
H82
H526
RPP
RPM
MFI of PD-L1
Control
AZD1775
D
MFI of PD-L1
siSCR
siWEE1
E
PD-L1+ cells / field
Vehicle
AZD1775
F
Vehicle
AZD1775
G
IFN-γ (ng/ml)
H82
H446
H526
0
0.5
5
PD-L1
H
H82
H526
H446
MFI of PD-L1
IFN-γ (ng/ml)
0 0.5 5

Figure S7. WEE1 inhibition increased IFN-γ mRNA and cell surface PD-L1 expression in SCLC, Related to Figure 5.

(A) Quantitative mRNA expression of *IFN-γ* in H82, RPP and RPM cells following knockdown of WEE1 with WEE1-specific siRNAs (#1 and #2) or nonspecific siRNA control. The bars represent mean ± SD of triplicate. Statistical significance was determined using Student's t-test (*$p < 0.05$, **$p < 0.01$). (B) The quantification of mean fluorescence intensity (MFI) of PD-L1 in SCLC cell (DMS114, H196, H211, H446, H526, H69, H82 and L311) by flow cytometry following 72 hour treatment with 1μM AZD1775. The bars represent mean of triplicate. (C) Representative MFI changes of PD-L1 expression in SCLC (H82, H526, RPP, and RPM) by flow cytometry following 72 hour treatment with 1 μM AZD1775. The bars represent mean ± SD of triplicate. Statistical significance was determined using Student's t-test (*$p < 0.05$, **$p < 0.01$). (D) MFI of PD-L1 expression in H82 cells following knockdown of WEE1 with specific siRNA or nonspecific siRNA control. The bars represent mean ± SD of triplicate. Statistical significance was determined using Student's t-test (**$p < 0.01$). (E) The number of PD-L1 expressed cells in immunohistochemistry staining that was performed in resected tumors of RPP model on day 21 (from Fig 3A). The average number of PD-L1 positive cells from five areas of five tumors in each group, and (F) the representative images of immunohistochemistry staining are shown. The data shown represent the means of five areas ± SD. Scale bar; 100μm. Statistical significance was determined using Student's t-test (***$p < 0.001$). (G) Representative histogram of PD-L1 expression in SCLC cells tested by flow cytometry following 48 hours treatment with 0.5 or 5 ng/ml of IFN-γ and (H) MFI of PD-L1 in SCLC (H82, H526, H446) treated with 0.5 or 5 ng/ml of IFN-γ. The bars represent mean ± SD of triplicate. Statistical significance was determined using Student's t-test (***$p < 0.001$).

Figure S8:

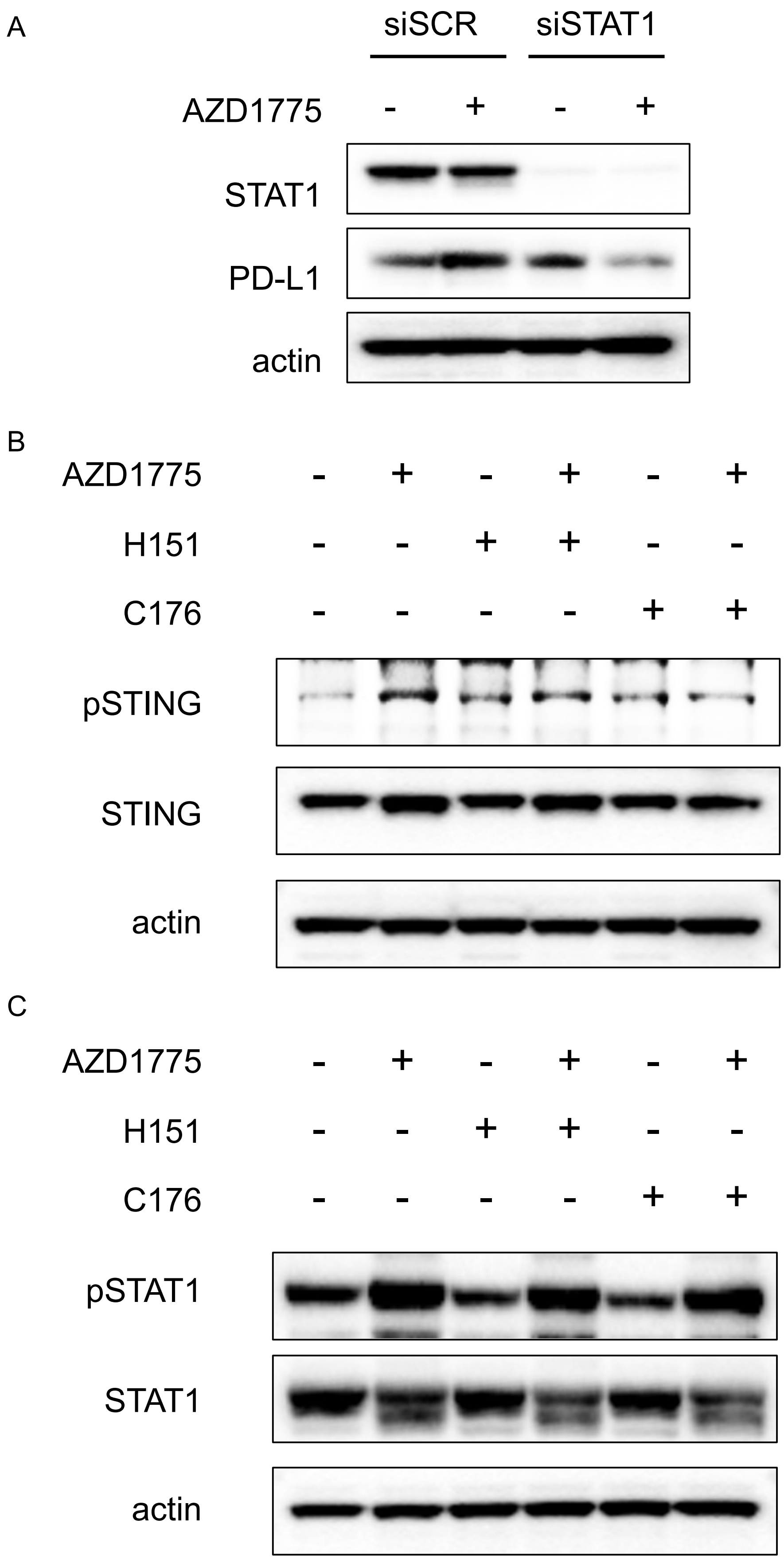
A
siSCR
siSTAT1
AZD1775 - + - +
STAT1
PD-L1
actin
B
AZD1775 - + - + - +
H151 - - + + - -
C176 - - - - + +
pSTING
STING
actin
C
AZD1775 - + - + - +
H151 - - + + - -
C176 - - - - + +
pSTAT1
STAT1
actin

Figure S8. Inhibition of STAT1 or STING with AZD1775 treatment in SCLC, Related to Figure 6.

(A) Knockdown of STAT1 (or nonspecific siRNA control [siSCR]) by siRNA followed by treatment with 0.5 μM AZD1775 for 72 hours in H82 cells. Western blot shows STAT1, PD-L1 and actin in H82 cells. (B)(C) Western blot shows (B) phospho (p)- and total (t)-STING in H526 treated with 1 μM AZD1775 and/or 0.5μM H151, 1μM C176 for 48 hours and (C) phospho (p)- and total (t)-STAT1 and actin in H526 treated with 1 μM AZD1775 and/or 0.5 μM H151, 1μM C176 for 24hours.

Table S1. Probes used for real time PCR, Related to STAR Methods.

Human

Gene	Company	Assay ID
IFN-α	Thermo Fisher	Hs03044218_g1
IFN-β	Thermo Fisher	Hs01077958_s1
IFN-γ	Thermo Fisher	Hs00989291_m1
CXCL10	Thermo Fisher	Hs00171042_m1
CCL5	Thermo Fisher	Hs99999048_m1
IRF1	Thermo Fisher	Hs00971960_m1
GAPDH	Thermo Fisher	Hs99999905_m1

Mouse

Gene	Company	Assay ID
IFN-α	Thermo Fisher	Mm00833969_s1
IFN-β	Thermo Fisher	Mm00439552_s1
IFN-γ	Thermo Fisher	Mm01168134_m1
CXCL10	Thermo Fisher	Mm00445235_m1
CCL5	Thermo Fisher	Mm01302427_m1
GAPDH	Thermo Fisher	Mm99999915_g1

Table S2. Antibodies used for flow cytometry, Related to STAR Methods.

Color	Maker	Company	Catalog#	Dilution
APC Cy7	CD4	BioLegend	100526	1/400
PE Cy7	CD8	BioLegend	100722	1/800
Pacific Blue	CD45	BioLegend	103126	1/100
PE-594	CD3	BioLegend	100246	1/100
Ghost violet 510	Live/Dead	Tonbo	13-0870-T500	1/100
BV605	PD1	BioLegend	135220	1/50
AF700	CD62L	BD	560517	1/50
BV711	CD44	BioLegend	103057	1/100
APC	TIM3	BioLegend	134008	1/200
BV711	GR-1	BioLegend	108443	1/200
APC	F4/80	Tonbo	20-4801-U100	1/100
BV650	CD11b	BioLegend	101259	1/100
PerCP Cy5.5	CD68	BioLegend	137010	1/100
PE	iNOS	Invitrogen	12-5920-80	1/50
PE	PD-L1 (murine)	BD	558091	1/100
APC	PD-L1 (human)	BioLegend	329708	1/100
FITC	H-2Kb/H-2Dd (murine)	BioLegend	114706	1/100
PE	HLA-ABC	BioLegend	311406	1/100

Table S3. Antibodies used for immunohistochemistry, Related to STAR Methods.

MARKER	EPITOPE RETRIEVAL	PRIMARY ANTIBODY VENDOR, CAT. NO.	PRIMARY ANTIBODY DILUTION	SECONDARY ANTIBODY SOURCE, CAT. NO.	SECONDARY ANTIBODY CONCENTRATION
**CD3**	Heat Induced, pH6	Abcam, ab135372	1:250	Leica Biosystems, DS9800 kit, reagent #3	Used at concentration provided by vendor
**CD8a**	Heat induced, pH9	Cedarlane, HS-361 003(Sy)	1:250	Leica Biosystems, DS9800 kit, reagent #3	Used at concentration provided by vendor
**PD-L1**	Heat induced, pH9	R&D System, AF1019	1:100	Vector Labs, BA5000	1:1000
